# Thermal and oxidative stability of the *Ocimum basilicum* L. essential oil/β-cyclodextrin supramolecular system

**DOI:** 10.3762/bjoc.10.298

**Published:** 2014-11-28

**Authors:** Daniel I Hădărugă, Nicoleta G Hădărugă, Corina I Costescu, Ioan David, Alexandra T Gruia

**Affiliations:** 1 Department of Applied Chemistry, Organic and Natural Compounds Engineering, Polytechnic University of Timişoara, Carol Telbisz 6, 300001, Timişoara, Romania; 2 Department of Food Science, Banat’s University of Agricultural Sciences and Veterinary Medicine “King Michael I of Romania” – Timişoara, Calea Aradului 119, 300645, Timişoara, Romania; 3 Regional Centre for Immunology and Transplant, County Clinical Emergency Hospital Timişoara, Iosif Bulbuca Blvd. 10, 300736, Timişoara, Romania

**Keywords:** basil, β-cyclodextrin, GC–MS analysis, nanoencapsulation, *Ocimum basilicum* L. essential oil, thermal and oxidative stability

## Abstract

*Ocimum basilicum* L. essential oil and its β-cyclodextrin (β-CD) complex have been investigated with respect to their stability against the degradative action of air/oxygen and temperature. This supramolecular system was obtained by a crystallization method in order to achieve the equilibrium of complexed–uncomplexed volatile compounds in an ethanol/water solution at 50 °C. Both the raw essential oil and its β-CD complex have been subjected to thermal and oxidative degradation conditions in order to evaluate the protective capacity of β-CD. The relative concentration of the *O. basilicum* L. essential oil compounds, as determined by GC–MS, varies accordingly with their sensitivity to the thermal and/or oxidative degradation conditions imposed. Furthermore, the relative concentration of the volatile *O. basilicum* L. compounds found in the β-CD complex is quite different in comparison with the raw material. An increase of the relative concentration of linalool oxide from 0.3% to 1.1%, in addition to many sesquiterpene oxides, has been observed. β-CD complexation of the *O. basilicum* essential oil modifies the relative concentration of the encapsulated volatile compounds. Thus, linalool was better encapsulated in β-CD, while methylchavicol (estragole) was encapsulated in β-CD at a concentration close to that of the raw essential oil. Higher relative concentrations from the degradation of the oxygenated compounds such as linalool oxide and aromadendren oxide were determined in the raw *O. basilicum* L. essential oil in comparison with the corresponding β-CD complex. For the first time, the protective capability of natural β-CD for labile basil essential oil compounds has been demonstrated.

## Introduction

Aromatic and pharmaceutical plants have been used in both food and medicinal fields since ancient time. These valuable plants have been used directly as raw materials (such as teas/infusions, ground spices, dressings, sauces, etc.) [1–3], or indirectly by isolation of the corresponding biologically active compounds. These were used as raw mixtures (e.g., extracts for food supplements), as well as purified bioactive compounds for the treatment of various diseases. They were used in natural form or after derivatization to a more bioactive compound, such as the flavonolignan silybin from *Silybum marianum* L. or its disuccinate derivative, with hepatoprotective properties [4–5].

*Ocimum* genus (Lamiaceae family) is comprised of more than fifty species of herbs and shrubs growing in tropical and warm temperate regions [6–9]. All of these plants contain various concentrations of volatile compounds (essential oils or volatile oils), among other compounds (e.g., flavonoids) [10], which furnish different flavoring and medicinal properties, such as antimicrobial, anti-inflammatory, analgesic, or antiseptic properties [11–14], in addition to antifungal and insect repellent properties [8,15]. By far, *Ocimum basilicum* L. (basil or sweet basil) is the most well-known herb from this class all over the world [9,16]. It originates from India and is comprised of many varieties such as Thai basil (var. *thyrsiflorum*) or lemon basil (var. *citriodora*) [17]. All of these varieties are mainly used fresh as salad and as seasoning (in sauces), or in drinks [18–19]. Traditionally, basil has been used for the treatment of stress, asthma, and diabetes [10,20]. Several studies have been performed to better understand its anti-oxidant [21–23], antiviral [10] medicinal properties, and its antimicrobial [19] biological effects. Moreover, the anticancer potential of basil components has been studied [24]. These sensory and pharmaceutical properties of basil are primarily due to the presence of essential oil components, which consist of monoterpene hydrocarbons (e.g., β-pinene), oxygenated monoterpenes (eucalyptol, linalool, α-terpineol, borneol and its acetate), phenolic derivatives (eugenol, methylchavicol (estragole)), sesquiterpene hydrocarbons (β-cubebene, β-caryophyllene, germacrene, etc.), and oxygenated sesquiterpenes (cadinol, spathulenol) [9,16,20,25].

During the processing, transportation, storage and even consumption of products containing such essential oils, some of thermal and/or oxidative labile constituents can be degraded to the point that they are ineffective, or even dangerous, toxic derivatives can be formed. There are many examples related to these aspects, such as diepoxidation of limonene to the carcinogenic diepoxylimonene or oxidation of other terpenes (e.g., pinene) to harmful oxidized derivatives [14,26–27], the degradation of safrole to carcinogenic metabolites [28], or the formation of oxygentated derivatives of limonene, linalool or caryophyllene, with allergenic and skin sensitization properties [29].

The formation of these harmful oxi-derivatives is supported by the access of oxygen/air to the reaction site of the terpene molecule. This reaction could be significantly reduced by molecular encapsulation of the bioactive compounds. One of the most frequently used matrices for molecular encapsulation, protection against oxidation or other degradation processes, as well as controlled release of relatively small bioactive compounds, are cyclodextrins (CDs) [30–35]. These are cyclic oligosaccharides consisting of α-(1→4)-linked glucopyranose units, which are obtained by starch degradation under controlled enzymatic conditions [36]. The most relevant natural CDs are α-, β-, and γ-CD, having six, seven, or eight glucopyranose moieties, but many other CD derivatives can be obtained in order to enhance their properties (e.g., aqueous solubility) [37–38]. The three-dimensional molecular structure of CDs looks like a truncated cone or cylinder, having a hydrophobic inner cavity and hydrophilic exterior bearing hydroxy groups [30,32]. This particular structure allows CDs to molecularly encapsulate relatively small hydrophobic molecules and allow for an increased apparent aqueous solubility of nanoencapsulated bioactive compounds. Moreover, the access of oxygen or other oxidative agents to these bioactive compounds can be reduced. Essential oil components are some of the most appropriate molecules for nanoencapsulation in CDs from both a geometrical and a hydrophobic point of view. The essential oil components (including basil essential oil), such as pinenes, linalool, terpineol and phenol derivatives can be appropriately nanoencapsulated in CDs due to their structural and hydrophobic characteristics [39–45]. The main compounds from *O. basilicum* L. essential oil, linalool and methylchavicol, have been used as guests for bicomponent encapsulation in CDs [39,41,46–47]. Linalool was encapsulated in β-CD [41] and its derivative 2-hydroxypropyl-β-CD [39] with good results and the complex formation was determined by thermal analysis (TG, DSC), FTIR and XRD. The quality of the encapsulation has been studied by gas chromatographic methods. Methylchavicol and other structurally related volatiles were also encapsulated in various natural CDs such as α- and β-CD, as well as in semisynthetic derivatives (2-hydroxypropyl-β-CD, randomly and low methylated β-CD) with good formation constants of almost 1000 M^–1^ for β-CD [46].

In this study, the evaluation of the protection capacity of natural β-CD for the main labile compounds of basil (*Ocimum basilicum* L.) essential oil under thermal and oxidative conditions using gas chromatography–mass spectrometry (GC–MS). The occurrence of harmful degradation compounds in raw basil essential oil and in the recovered product of the β-CD complex subjected to thermal/oxidative degradation has been comparatively evaluated.

## Results and Discussion

### *Ocimum basilicum L.* essential oil composition

**Figure 1:**
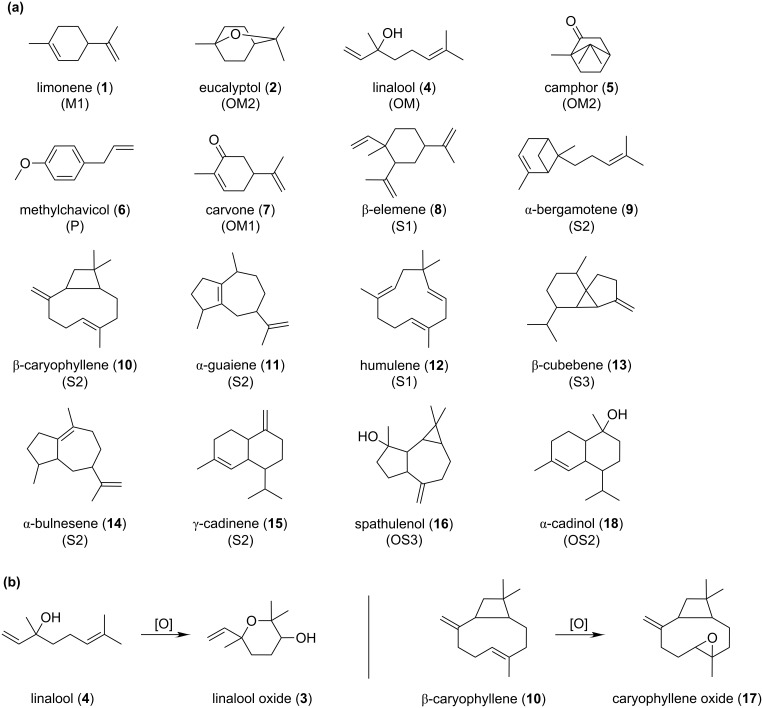
The main compounds identified in raw *O. basilicum* L. essential oils (a) and the degradation reactions for some labile compounds (b). The number given for each compound can correspondingly be found in Table 1. The compound class is indicated in parenthesis (M – acyclic monoterpenes, OM – oxygenated acyclic monoterpenes, OM1/2 – oxygenated mono/bicyclic monoterpenes, P – phenolic derivatives, S1/2/3 – mono/bi/tricyclic sesquiterpenes, OS2/3 – oxygenated bi/tricyclic sesquiterpenes).

*Ocimum basilicum* L. essential oil contains many volatile compounds comprised of monoterpenes, oxygenated monoterpenes, sesquiterpenes and their oxygenated derivatives, as well as phenolic derivatives (Figure 1 and Table 1). The most important constituents were linalool (**4**) (27.8%) and methylchavicol (**6**) (19.6%), from oxygenated monoterpene and phenolic derivative compound classes, respectively. Limonene (**1**) was the most important component from the monoterpene class (0.5%), while other monoterpene derivatives in the *O. basilicum* L. essential oil were eucalyptol (**2**), camphor (**5**) and carvone (**7**). On the other hand, sesquiterpenes were more abundant in the *O. basilicum* L. essential oil samples in comparison with their oxygenated derivatives. The quantified sesquiterpenes were 17.1%, while the oxygenated sesquiterpenes were in a relative concentration of 8.4%. The most important compounds in the first case were β-elemene (**8**) (4.6%), γ-cadinene (**15**) (4.1%), α-bulnesene (**14**) (2.1%), α-guaiene (**11**) (1.6%), β-cubebene (**13**) (1.5%), α-bergamotene (**9**) (1.2%), humulene (**12**) (1.2%), and caryophyllene (**10**) (0.9%). α-Cadinol (**18**) (6.2%) and spathulenol (**16**) (2.2%) were the most important sesquiterpene derivatives in the *O. basilicum* L. essential oil. Some of degradation compounds (mainly a result of the separation process) can also be identified in the raw *O. basilicum* L. essential oil sample, such as caryophyllene oxide (**17**) (0.4%), aristolene oxide (**21**) (0.3%), aromadendrene oxide (**23**) and isoaromadendrene oxide (**22**) (up to 0.2%), or the corresponding diols (up to 0.1%).

**Table 1:** The GC–MS data (Kovats index – KI, relative concentrations – Area (%)) of the raw (B) and recovered from β-CD complex (B/β-CD) of *O. basilicum* L. essential oil, the relative encapsulation efficiency (E.E.) and the log P values.

Entry	KI	MS Identification	Area (%) (B)^a^	Area (%) (B/β-CD)^a^	Relative E.E. (%)^b^	log P^c^

1	1085	Limonene	0.54	0.43	−20.4	3.615
2	1106	Eucalyptol	1.76	1.41	−19.9	2.716
3	1149	Linalool oxide	0.31	0.06	−80.6	1.942
4	1182	Linalool	27.82	42.48	52.7	3.213
5	1279	Camphor	1.80	1.17	−35.0	2.160
6	1337	Methylchavicol	19.64	22.52	14.7	2.818
7	1393	Carvone	2.33	1.77	−24.0	2.513
8	1460	β-Elemene	4.60	4.08	−11.3	5.369
9	1497	α-Bergamotene	1.16	0.94	−19.0	5.239
10	1503	β-Caryophyllene	0.94	0.81	−13.8	5.174
11	1509	α-Guaiene	1.55	1.28	−17.4	4.890
12	1547	Humulene	1.17	0.86	−26.5	5.305
13	1578	β-Cubebene	1.46	1.07	−26.7	5.609
14	1598	α-Bulnesene	2.11	1.75	−17.1	5.071
15	1617	γ-Cadinene	4.09	3.29	−19.6	5.755
16	1720	Spathulenol	2.19	1.43	−34.7	3.909
17	1727	Caryophyllene oxide	0.35	0.20	−42.9	4.136
18	1782	α-Cadinol	6.21	0.96	−84.5	4.974
19	1794	Sesquiterpene oxide (isomer 1)^d^	0.09	Tr.^e^	−	−
20	1851	Ionone oxide	Tr.^e^	0.09	−	−
21	1896	Aristolene epoxide	0.27	0.13	−51.9	4.271
22	1910	Isoaromadendrene epoxide	0.06	Tr.^e^	−100.0	4.267
23	1932	Aromadendrene oxide	0.13	0.05	−61.5	4.175
24	1986	Alloaromadendrene oxide	0.05	−	−	−
25	2036	Sesquiterpene oxide (isomer 2)^d^	0.09	−	−	−
26	2066	Spiro[tricyclo[4.4.0.0(5,9)]decane-10,2'-oxirane], 1-methyl-4-isopropyl-7,8-dihydroxy	0.05	−	−	−
27	2105	Sesquiterpene oxide (isomer 3)^d^	0.06	−	−	−
28	2128	6-Isopropenyl-4,8a-dimethyl-1,2,3,5,6,7,8,8a-octahydronaphthalene-2,3-diol	0.08	−	−	−
29	2148	Sesquiterpene oxide (isomer 4)^d^	0.05	−	−	−
		Other minor compounds	19.04	13.22		

^a^B – Basil (*O. basilicum* L.) essential oil; B/β-CD – recovered basil (*O. basilicum* L.) essential oil from the β-CD complex; ^b^E.E. – encapsulation efficiency, calculated as the percent enhancement the relative concentration of the compound (which can be positive or negative) relative to the relative concentration in the raw essential oil; ^c^logarithm of the octanol/water partition coefficient, calculated according to: http://www.molinspiration.com/cgi-bin/properties (Molinspiration Cheminformatics); ^d^unidentified sesquiterpene oxides; ^e^concentrations lower than 0.04%.

### Obtaining of *O. basilicum L.* essential oil/β-CD complex

The *O. basilicum* L. essential oil/β-CD complex was obtained by using the controlled crystallization method from an ethanol/water suspension (due to the lower water solubility, even at higher temperatures). This method allows the equilibrium between the uncomplexed and complexed compounds from the essential oil to occur, including the molecular competitivity in the encapsulation process. However, the essential oil/β-CD complex is generally less soluble in the ethanol/water solution than the unfilled β-CD. As a result, a well-formed complex could be obtained by using this method. Furthermore, this essential oil/β-CD complex could be appropriately evaluated from a degradative stability point of view. The degradation assumes oxygen/air as an environmental medium, which has a lower solubility (especially at higher temperature) as compared to the ethanol/water mixture environment present during the complexation process.

The complexation yield, expressed as the ratio between the essential oil/β-CD complex mass and the masses of the essential oil and β-CD used in the complexation process, was 74.2%. This relatively high recovery yield for the β-CD complex can be explained by the lower water (as well as ethanol/water) solubility. The quality of the essential oil/β-CD complex formation was sustained by the morphology of the complex crystals, evaluated by scanning electron microscopy, SEM (Inspect S, voltage of 25 kV, magnification of 3000–12000×, focus of 10–14.1 mm; see Supporting Information File 1). Rhombohedral prisms with dimensions up to ten micrometers for *O. basilicum* L. essential oil/β-CD complex were obtained.

The relative concentration of the main compounds of the *O. basilicum* L. essential oil varies greatly in β-CD complexes in comparison with those from the raw sample. To this effect, linalool (**4**) was better encapsulated than sesquiterpenes (especially sesquiterpene alcohols), in comparison with the case of raw *O. basilicum* L. essential oil. In the raw sample, linalool (**4**) was found at a relative concentration of 27.8%. In the recovered essential oil from the complex, the relative concentration of this compound was higher at 42.5%. An increase in the relative concentration of almost 53% was observed. This increase/decrease (which can be positive or negative) is calculated as the percent of the ratio between the difference in the relative concentrations for the degraded and the raw samples and the concentration in the raw essential oil. The same behavior was observed for the aromatic compound methylchavicol (**6**), which was better encapsulated in β-CD in comparison with the raw essential oil (relative concentration of 19.6% in the raw sample, in comparison with 22.5% for the recovered essential oil, with a level of increase of 14.7%, Table 1). This enhanced relative concentration of linalool (**4**) and methylchavicol (**6**) in the β-CD complex is primarily due to their high concentrations in the raw essential oil, which allows for better molecular encapsulation in the CD complexation process. Furthermore, the relatively small dimensions of these molecules (especially for the highly flexible linalool (**4**) molecule) in comparison with competitive molecules (more bulky cyclic mono- and sesquiterpenoids) play an important role for encapsulation competitivity. However, the most concentrated sesquiterpene alcohol in the raw essential oil (α-cadinol (**18**), 6.2%) was more weakly encapsulated in β-CD (approximately 1%), likely due to its rigid structure and lower hydrophobicity in comparison with other sesquiterpenoids. The same observation was made for spathulenol (**16**), which had relative concentrations of 2.2% and 1.4%, respectively. Most of the other compounds were encapsulated at similar relative concentrations. Generally, oxygenated monoterpenes were encapsulated at a lower relative concentration than the corresponding monoterpenes. For example, limonene (**1**) was encapsulated at only 20% lower than camphor (**5**) and carvone (**7**), having relatively reduced concentrations in the encapsulated essential oil of 35% and 24%, respectively. Methylchavicol (**6**), a phenolic derivative, is also better encapsulated in β-CD due to its rigid and hydrophobic structure (log P = 2.8), although it is an oxygenated compound. The ether structure renders methylchavicol (**6**) more similar to limonene (**1**), which has a relatively rigid and hydrophobic structure. The same behavior for sesquiterpenes (hydrocarbons), in comparison with the corresponding oxygenated sesquiterpenes exists. The above mentioned α-cadinol (**18**) and spathulenol (**16**) were identified in the encapsulated essential oil at reduced concentrations of 34.5% and 84.5%, respectively. This reduction in concentration is more significant than for the case of the corresponding hydrocarbons. Lower relative concentrations in the encapsulated essential oil were reduced to 19.6% for γ-cadinene (**15**), 17.4% for α-guaiene (**11**) and 17.1% for α-bulnesene (**14**), relative to a relative concentration in the raw essential oil of 4.1% to 3.3%. In the recovered oil, reduced concentrations of 1.55% to 1.3%, and 2.1% to 1.75% were observed (see Table 1). Furthermore, all terpene epoxides (even those found in lower relative concentrations) were encapsulated in very low concentrations in the β-CD complexes in comparison with the raw *O. basilicum* L. essential oil. This feature can be observed for the relative concentrations in the complex with an increase of 81% for linalool epoxide (**3**), 43% for caryophyllene epoxide (**17**), 52% for aristolene epoxide (**21**) and 61.5% for aromadendrene epoxide (**23**) (Table 1).

The hydrophobicity and structural characteristics play an important role for both encapsulation efficiency and competitivity. Thus, a statistically significant correlation could be obtained for the encapsulation efficiency (E.E.) of oxygenated monoterpenes if the hydrophobicity (log P) is considered as the parameter as:

[1]
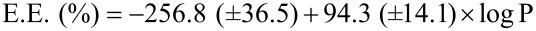


where *n* = 6, *r* = 0.958, *s* = 14.5, *F* = 45. On the other hand, the hydrophobicity does not correlate in the case of larger molecules such as sesquiterpenes and their oxygenated derivatives. The steric hindrance is likely the most important aspect related to both encapsulation efficiency and competitivity in the molecular encapsulation process.

The competition for nanoencapsulation of *O. basilicum* L. essential oil constituents in β-CD is better assessed by using multivariate statistical analysis (PCA) for both the encapsulation data and the structural descriptors. All essential oil compounds were classified taking into account the terpenoid structure (mono- and sesquiterpenoid hydrocarbons (M and S), oxygenated mono- and sesquiterpenes (OM and OS), as well as the cycle numbers (1/2/3/4 included in the sample code), which indicates the structure rigidity/flexibility). The PCA variables are the relative encapsulation efficiency and the hydrophobicity (log P). These two variables include both structural characteristics (from E.E.) and transport/van der Waals interaction aspects (from log P). These parameters retain characteristics from one another. The PCA analysis allows extraction of the useful information from the non-orthogonal parameters to the orthogonal ones, principal components, *PC*_1_ and *PC*_2_. In principal, the translation and rotation of the initial axes in order to obtain maximum variance for every *PC*, with respect to the orthogonality, must be performed. The translation coordinates are related to a score plot that associates similarities between structures. The axis rotation parameters provide a loading plot, which indicates the importance of the parameters for this analysis.

**Figure 2:**
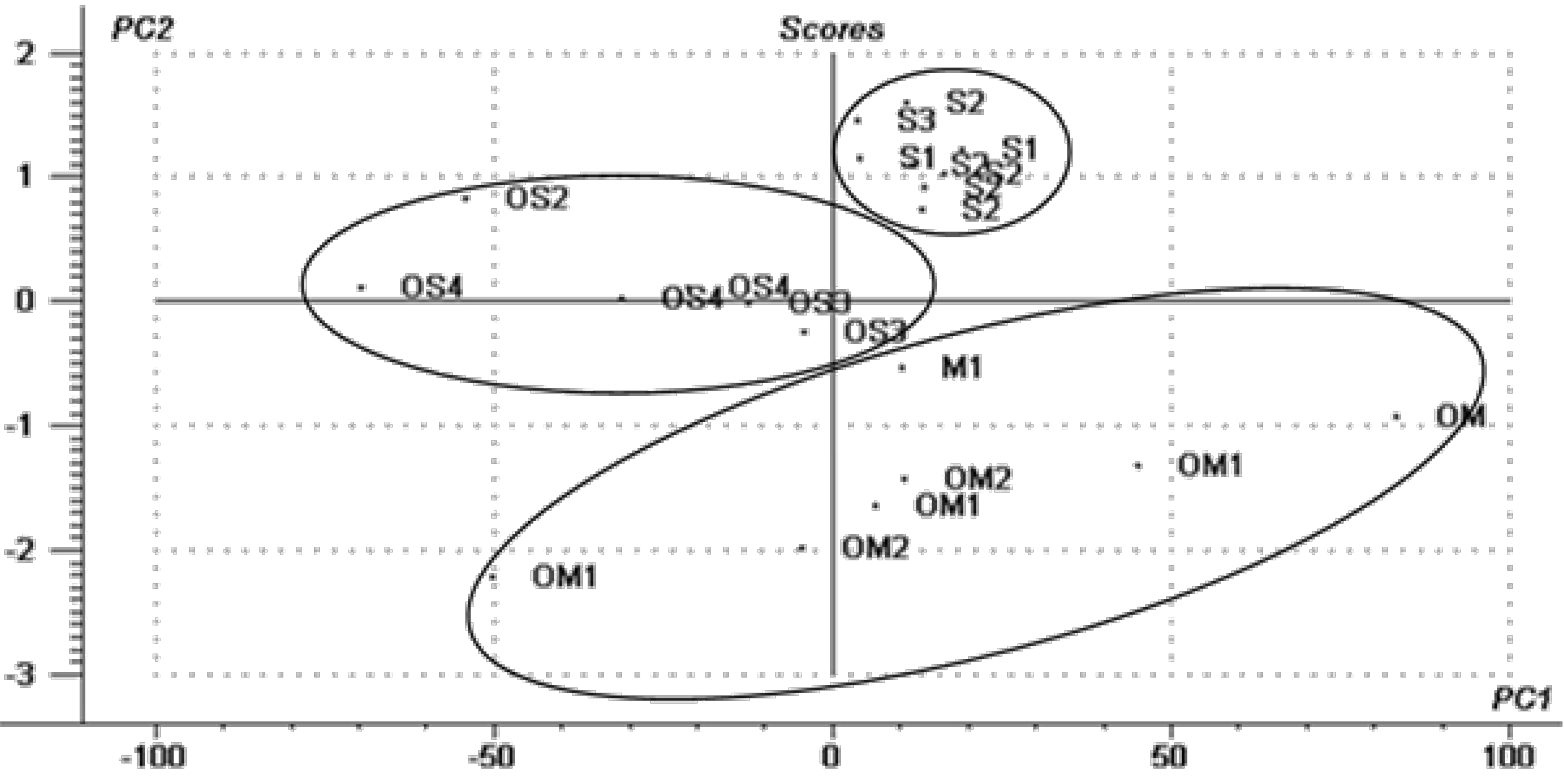
The score plot from the PCA analysis of the *O. basilicum* L. essential oil compounds nanoencapsulation in β-CD (classification as acyclic or cyclic (numbered) mono- and sesquiterpenoid hydrocarbons (M and S), or the corresponding oxygenated terpenes (OM and OS); the encapsulation efficiency and hydrophobicity were the PCA variables).

The bi-dimensional PCA analysis clearly classifies the sesquiterpenoid hydrocarbons in the upper region of the PCA score plot. Oxygenated sesquiterpenes are located in the middle-left region of this plot and the number of cycles seems to have no influence. On the other hand, monoterpenoids (both hydrocarbonated and oxygenated derivatives) were classified in the lower part of this plot (Figure 2). The hydrophobicity (log P) of the compounds is responsible for the classification of compounds by *PC*_1_, while the encapsulation efficiency is especially responsible for classification by *PC*_2_. The bi-dimensional statistical analysis of the β-CD nanoencapsulation of *O. basilicum* L. essential oil compounds clearly reveals the importance of hydrophobicity, steric fit and flexibility for the encapsulation competitivity (see Supporting Information File 1 for the PCA variable influence).

### Degradation of *O. basilicum L.* essential oil

The thermal/oxidative degradation studies were performed in sealed flasks, under air at normal humidity, by maintaining the samples two hours at various temperatures (50 °C, 100 °C, and 150 °C). The diluted samples were analyzed by GC–MS in order to evaluate the variation of the relative concentration of the main *O. basilicum* L. essential oil compounds and to identify the possible harmful degradation compounds, which can result from oxidative reactions at high temperatures. The relative concentrations of the original compounds as well as some of the degraded compounds as a result of oxidation are presented in Table 2. An increase of 3–4% in the relative concentration for the main compounds was observed. Thus, the concentration of linalool (**4**) increases from almost 28% in the raw sample to 31–32% in the degraded essential oils, while the concentration of methylchavicol (**6**) increases from 19.6% in the raw oil to 21–22% in the degraded samples (Table 2). Other oxygenated monoterpenes were found only slightly more concentrated in the degraded samples than in raw essential oil (camphor (**5**), with an increase from 1.8% to 2.1%). In contrast, the concentration of eucalyptol (**2**) decreases after degradation from 1.8% to 1.55%. An important variation of the relative concentration was found for the degradation compound linalool oxide (**3**), which is only 0.3% in the raw essential oil and increased to 1.1% at a high temperature degradation (an increase of 250%).

**Table 2:** The GC–MS data for the non-degraded (B) and thermal/oxidative degraded *O. basilicum* essential oil at various temperatures (B_50_ – degradation temperature of 50 °C, B_100_ – 100 °C, B_150_ – 150 °C).

Entry	KI	MS Identification	Area (%) (B)	Area (%) (B_50_)	Area (%) (B_100_)	Area (%) (B_150_)

1	1085	Limonene	0.54	0.68	0.48	0.77
2	1106	Eucalyptol	1.76	1.44	1.43	1.55
3	1149	Linalool oxide	0.31	0.22	0.25	1.09
4	1182	Linalool	27.82	32.32	31.76	31.00
5	1279	Camphor	1.80	1.55	1.62	2.07
6	1337	Methylchavicol	19.64	20.90	21.56	22.01
7	1393	Carvone	2.33	2.05	2.08	2.34
8	1460	β-Elemene	4.60	4.31	4.44	4.86
9	1497	α-Bergamotene	1.16	1.02	1.04	1.01
10	1503	β-Caryophyllene	0.94	0.84	0.87	0.34
11	1509	α-Guaiene	1.55	1.36	1.39	0.98
12	1547	Humulene	1.17	0.96	0.94	0.65
13	1578	β-Cubebene	1.46	1.25	1.12	0.10
14	1598	α-Bulnesene	2.11	1.77	1.82	1.13
15	1617	γ-Cadinene	4.09	3.66	3.73	3.77
16	1720	Spathulenol	2.19	1.94	1.96	1.90
17	1727	Caryophyllene oxide	0.35	0.31	0.32	0.22
18	1782	α-Cadinol	6.21	5.90	5.85	5.78
19	1794	Sesquiterpene oxide (isomer 1)^a^	0.09	0.12	0.14	0.31
20	1851	Ionone oxide	–	0.12	0.14	0.31
21	1896	Aristolene epoxide	0.27	0.24	0.23	0.18
22	1910	Isoaromadendrene epoxide	0.06	0.04	0.04	0.06
23	1932	Aromadendrene oxide	0.13	0.11	0.12	0.16
24	1986	Alloaromadendrene oxide	0.05	0.04	0.04	0.29
25	2036	Sesquiterpene oxide (isomer 2)^a^	0.09	0.08	0.06	0.06
26	2066	Spiro[tricyclo[4.4.0.0(5,9)]decane-10,2'-oxirane], 1-methyl-4-isopropyl-7,8-dihydroxy	0.05	0.05	0.05	–
27	2105	Sesquiterpene oxide (isomer 3)^a^	0.06	0.07	0.05	–
28	2128	6-Isopropenyl-4,8a-dimethyl-1,2,3,5,6,7,8,8a-octahydronaphthalene-2,3-diol	0.08	0.08	0.07	0.15
29	2148	Sesquiterpene oxide (isomer 4)^a^	0.05	0.09	0.05	–
		Other minor compounds	19.04	16.48	16.35	16.91

^a^Unidentified sesquiterpene oxides.

Generally, sesquiterpene hydrocarbons were found to degrade in *O. basilicum* L. essential oil samples at lower relative concentrations that in the raw sample (Table 2). Important variations were observed for β-caryophyllene (**10**), α-guaiene (**11**), humulene (**12**), β-cubebene (**13**) and α-bulnesene (**14**) (a decrease of 37–93%), while α-bergamotene (**9**) and γ-cadinene (**15**) decrease by only 7.8–12.9% at a degradation temperature of 150 °C. In the case of β-elemene (**8**), an increase of 5.7% up to the degradation temperature of 150 °C was observed. Sesquiterpene alcohols were found in lower relative concentrations in the degraded samples than in the raw essential oil (spathulenol (**16**) and α-cadinol (**18**)).

The degradation compounds resulting from the severe degradation conditions imposed on the *O. basilicum* L. essential oil have a very different behavior. Thus, the most important variation was observed for alloaromadendrene oxide (**24**) (with a very large increase of 480%, relative to a relative concentration in the raw essential oil of 0.05% to 0.3% at the highest degradation temperature) and for an unidentified sesquiterpene oxide (with an increase of 244%, from 0.1% to 0.3%, Table 2). Other epoxy derivatives appear upon degradation, such as ionone oxide (**20**). This compound was found increasingly concentrated in degraded samples (and not identified in the raw sample). Some of sesquiterpene oxides found in the raw essential oil exhibited a slow decrease in the relative concentrations in the degraded samples (e.g., caryophyllene oxide (**17**) and aristolene epoxide (**21**)). This behavior suggests that the sesquiterpenes are more susceptible to degradation than the corresponding alcohols. The same argument can be applied for methylchavicol (**6**), which appears as a stable methoxy group in structure, as well as for linalool (**4**), and even the presence of its oxide (**3**) which is important in the degraded samples at very high degradation temperature.

### Degradation of *O. basilicum L.* essential oil/β-CD complex

In order to reduce the level of degradation occurring in *O. basilicum* L. essential oil during processing, transportation, storage and consumption, its nanoencapsulation in β-CD has been employed. The same degradation studies performed for the raw essential oil have also been performed for the *O. basilicum* L. essential oil/β-CD complexes. All of the volatile and degraded compounds from the β-CD complex were extracted into hexane by multiple liquid–solid extraction steps until no volatile compounds could be identified in the last extract. The relative concentrations of the complexed essential oil components as well as the degraded compounds were determined by GC–MS analysis (see Supporting Information File 1 for further information). No important variations in the relative concentrations of β-CD nanoencapsulated components or their degradation compounds were observed (Table 3). The concentration of the main compounds linalool (**4**) and methylchavicol (**6**) varied over the narrow ranges of 41.4–43% and 22.5–23.9%, respectively (percent increase of only 1.25% and 0.75%, respectively). The increase for the other monoterpenoids varied up to 10% (for camphor (**5**)), but generally in the range of 5–7% (almost 5% for limonene (**1**) and carvone (**7**), and 7% for eucalyptol (**2**)). The increase of the relative concentration of the degradation compound linalool oxide (**3**) was significantly reduced by nanoencapsulation in β-CD; this concentration value is only 0.1% even at a high degradation temperature (an of 83%, in comparison with 250% for non-encapsulated essential oil samples, Table 2 and Table 3).

**Table 3:** The GC–MS data for the *O. basilicum* essential oil recovered from the non-degraded and thermal/oxidative degraded β-CD complex (at various temperatures; B/β-CD – non-degraded *O. basilicum* L. essential oil/β-CD complex, B/β-CD_50,100,150_ – degraded *O. basilicum* L. essential oil/β-CD complex at a degradation temperatures of 50 °C, 100 °C, and 150 °C, respectively). The numbers for the compounds correspond to those from Table 1 and Table 2.

Entry	KI	MS Identification	Area (%) (B/β-CD)	Area (%) (B/β-CD_50_)	Area (%) (B/β-CD_100_)	Area (%) (B/β-CD_150_)

1	1085	Limonene	0.43	0.48	0.45	0.45
2	1106	Eucalyptol	1.41	1.43	1.44	1.51
3	1149	Linalool oxide	0.06	0.08	0.04	0.11
4	1182	Linalool	42.48	42.23	41.42	43.01
5	1279	Camphor	1.17	1.18	1.09	1.29
6	1337	Methylchavicol	22.52	22.82	23.89	22.69
7	1393	Carvone	1.77	1.81	1.83	1.86
8	1460	β-Elemene	4.08	4.20	4.43	4.52
9	1497	α-Bergamotene	0.94	0.95	1.01	1.02
10	1503	β-Caryophyllene	0.81	0.82	0.87	0.84
11	1509	α-Guaiene	1.28	1.33	1.40	1.34
12	1547	Humulene	0.86	0.88	0.93	0.96
13	1578	β-Cubebene	1.07	1.05	1.11	0.82
14	1598	α-Bulnesene	1.75	1.80	1.89	1.73
15	1617	γ-Cadinene	3.29	3.37	3.59	3.39
16	1720	Spathulenol	1.43	1.49	1.49	1.47
17	1727	Caryophyllene oxide	0.20	0.21	0.17	0.21
18	1782	α-Cadinol	0.96	0.92	0.92	0.74
20	1851	Ionone oxide	0.09	0.12	0.11	0.13
21	1896	Aristolene epoxide	0.13	0.14	0.12	0.13
23	1932	Aromadendrene oxide	0.05	0.05	0.04	0.05
		Other minor compounds	13.22	12.64	11.76	11.73

Similar behavior was observed for the case of sesquiterpenoids. An increase of 2.8–11.6% was observed for most of the sesquiterpene hydrocarbons and alcohols. For example, β-elemene (**8**) had an increase in the relative concentration from 4.1% in the non-degraded *O. basilicum* L. essential oil/β-CD complex to 4.5% in the degraded one, α-bergamotene (**9**) from 0.9% to 1%, β-caryophyllene (**10**) from 0.81% to 0.84%, α-guaiene (**11**) from 1.28% to 1.34%, humulene (**12**) from 0.86% to 0.96%, γ-cadinene (**15**) from 3.3% to 3.4%, and spathulenol (**16**) was basically unchanged (1.43–1.49%). Few compounds exhibited a decrease in the relative concentration during the degradation. This decrease was observed for the case of β-cubebene (**13**) from 1.07% to 0.82% (a decrease of 23.4%), α-cadinol (**18**) from 0.96% to 0.84% (a decrease of almost 23%) and α-bulnesene (**14**) with a decrease of only 1.1% (Table 3).

The degradation compounds from the sesquiterpene epoxide class were found in very low relative concentrations in the recovered *O. basilicum* L. essential oil from the non-degraded or degraded β-CD complex. Caryophyllene oxide (**17**) had a relative concentration in the non-degraded or degraded encapsulated essential oil of 0.2%, aristolene epoxide (**21**) 0.12–0.14%, aromadendrene oxide (**23**) 0.04–0.05%, and ionone oxide (**20**) 0.09–0.13%, in comparison with the non-encapsulated essential oil, where these concentrations were much larger (see Table 2 and Table 3). Furthermore, some of the degradation compounds identified in the degraded raw essential oil were not found in the recovered essential oil from the degraded β-CD complex (isoaromadendrene epoxide (**22**), alloaromadendrene oxide (**24**), and other unidentified sesquiterpene oxides).

The protection capacity of β-CD against degradation of labile compounds from *O. basilicum* L. essential oil was revealed by the increase in the degradation compounds as a result of the oxidation reaction especially at higher temperatures (Figure 1). Linalool oxide (**3**) had a relative concentration of more than three times that found in the degraded raw essential oil at 150 °C, while this concentration was not more than two times higher in the β-CD complex. Better protection was observed in the case of sesquiterpenoids. The oxidation of β-carryophyllene (**10**) to its epoxide (**17**) (Figure 1) corresponds to a lower relative concentration of the degradation compound in the β-CD complex. Significant protection against oxidation at higher temperatures was observed in the case of alloaromadendrene and in unidentified sesquiterpenes. Their epoxides were encapsulated in β-CD in lower relative concentrations, which enhances the quality of the *O. basilicum* L. essential oil/β-CD complex.

## Conclusion

In this study, the protective capacity of β-CD against oxidation at higher temperatures for the labile volatile components of *Ocimum basilicum* L. (sweet basil) essential oil has been demonstrated by GC–MS analysis. Indeed, the potentially harmful epoxidated mono- and sesquiterpenes (such as linalool oxide (**3**)), which appear at high concentrations in degraded raw essential oil, are limited by and almost constant in the β-CD complex, even at very high degradation temperatures. However, the competition of the main components of *O. basilicum* L. essential oil (due to their hydrophobicity and overall structural architecture) for nanoencapsulation in β-CD should be evaluated. The volatile compounds of interest from *O. basilicum* L. essential oil (e.g., linalool (**4**) and methylchavicol (**6**)) are better encapsulated in β-CD as compared to some of the sesquiterpenes and especially with respect to sesquiterpene epoxides (some of them are reactive and possibly harmful compounds), which indicates the enhanced quality and stability of the *O. basilicum* L./β-CD complex.

## Experimental

### Materials

The *Ocimum basilicum* L. (basil) essential oil was obtained from S.C. Natex S.A. Cluj Napoca (Romania) as a gift (the properties were: pale yellow, oily liquid, relative density of 0.90–0.92 at 20 °C and refractive index of 1.473–1.490 at 20 °C). It was stored in a refrigerator until analysis and complexation. The β-CD used for the *O. basilicum* L. essential oil nanoencapsulation was obtained from CycloLab, Budapest (purity >98%). Ethanol of 96% (v/v; Chimopar, Bucharest) was used in the complexation process and hexane (GC grade, Sigma-Aldrich) was used for recovering the volatile compounds from the β-CD complex and as solvent for the raw and degraded *O. basilicum* L. essential oil. Anhydrous calcium chloride (pro analysis) was purchased from Chimopar, Bucharest. The Kovats indices of the *O. basilicum* L. essential oil compounds and their degradation derivatives were obtained by using a C_8_–C_20_ alkane standard solution (Fluka Chemie AG).

### Methods

#### Synthesis of an *O. basilicum* L. essential oil/β-CD complex

The method used to obtain the essential oil/β-CD complex was the crystallization from a ethanol/water solution. Here, a mass of β-CD hydrate was suspended (due to the lower water solubility of β-CD) in distilled water at 50 ± 2 °C in a double-walled reactor (temperature controlled with a thermal-controlled water bath, Ultrathermostat IIM, Bucharest), equipped with a magnetic stirring system, a reflux condenser and a dropping funnel. The β-CD:*O. basilicum* L. essential oil molar ratio was 1:1. The water concentration of the β-CD hydrate was 12.4% (determined by loss upon drying method). The essential oil mass was calculated by taking into account the main volatile compounds identified and quantified by GC–MS (linalool (**4**) and methylchavicol (**6**) in a mass ratio of 1.4:1). The corresponding mass of essential oil was dissolved in 96% ethanol at a concentration of 15.3 mg/mL and was added to the β-CD suspension by the dropping funnel over the course of 15 minutes at 50 °C under continuous stirring. The formation of the complex was completed by continued stirring at the same temperature for another 30 minutes followed by slow crystallization by cooling to room temperature over the course of four hours. The cooling rate was approximately 7.5 °C/h. This was performed under magnetic stirring using the same thermal-controlled water bath. The *O. basilicum* L. essential oil/β-CD complex crystal formation was completed by refrigeration at 4 °C overnight. The complex crystals (containing both β-CD complex and non-complexed β-CD, which are very difficult to separate from one another) were filtered under vacuum, washed with ethanol and dried in a desiccator with anhydrous calcium chloride. The essential oil/β-CD complex was stored in sealed containers at 4 °C until the degradation study and physico-chemical analysis was performed. Only one sample of the complex of the natural β-CD was subjected to degradation. All data on the complexation process are shown in the Supporting Information File 1.

#### Degradation of the *O. basilicum* L. essential oil and its β-CD complex

Thermal and oxidative degradation of *O. basilicum* L. essential oil and its β-CD complexes were performed in a thermal-controlled oven in the presence of air at atmospheric pressure. The raw essential oil was weighed (approximately 50 mg) and uniformly distributed on the bottom of a glass flask with a surface area of 500 mm^2^. In the case of *O. basilicum* L. essential oil/β-CD complex, a mass of the solid sample containing approximately the same amount of essential oil (≈50 mg) was added in a similar flask. Both sample types (raw essential oil and the corresponding β-CD complex) were subjected to degradation in the sealed flasks in the presence of air at atmospheric pressure, relative humidity of 70% and temperatures of 50 °C, 100 °C, and 150 °C for two hours. After cooling, the degraded raw essential oil was recovered with hexane (4 × 1 mL) and the solution was dried over anhydrous calcium chloride and analyzed using GC–MS. In the case of the degraded *O. basilicum* L. essential oil/β-CD complex, the samples were dissolved or suspended in distilled water (4 × 2 mL) and extracted three times with hexane (3 × 2 mL) and refluxed for 10 minutes for every step in a thermostated extractor equipped with a condenser and a magnetic stirring system. The cooled hexane extracts were dried over anhydrous calcium chloride and analyzed using GC–MS.

#### GC–MS analysis of the raw and degraded *O. basilicum* L. essential oil (non-encapsulated and encapsulated in β-CD)

The analysis of the raw and recovered material from the β-CD complex *O. basilicum* L. essential oil, that is, the non-degraded or thermal/oxidative degraded samples, was performed by gas chromatography coupled with mass spectrometry (GC–MS). A GC Hewlett Packard HP 6890 Series gas chromatograph connected to a Hewlett Packard 5973 Mass Selective Detector was used. The following GC working conditions were used: HP-5 MS column (length 30 m, inner diameter 0.25 mm, and film thickness 0.25 μm), temperature program of 50 °C to 250 °C with a heating rate of 6 °C/min, both injector and detector temperatures of 280 °C, helium as carrier gas, and an injection volume of 2 μL. An EI energy of 70 eV, a source temperature of 150 °C, a scan range of 50–300 amu, and a scan rate of 1 s^−1^ were used for the MS system. The relative concentrations of the *O. basilicum* L. essential oil components were calculated as mass percent by using the area normalization method (the relative concentration of the compound is assumed equal to the corresponding percent of the GC area divided by the total area of compounds). The acquisition and handling of the GC–MS data was performed by using the Enhanced MSD ChemStation version D.02.00.275/2005 package (Agilent Technologies).

Two methods of identification of the *O. basilicum* L. essential oil constituents (both from the raw or recovered from the β-CD complexes, non-degraded or thermal degraded) were used. The first method assumes the comparison of the experimental MS spectra with those from the NIST/EPA/NIH Mass Spectral Library 2.0 (2002). The second method involves comparison of the calculated Kovats index (KI) for some compounds with those previously determined by our research group. The KIs were obtained by interpolating the retention time (*t*_R_) of the compound in the KI versus *t*_R_ graph obtained for the C_8_–C_20_ alkane standard mixture analyzed under the same conditions by GC–MS.

#### Statistical analysis

The β-CD encapsulation-competition data and the main descriptor indicating the hydrophobicity of the compounds (log P – the logarithm of 1-octanol/water partition coefficient), which were used in the statistical multivariate analysis, were calculated according to http://www.molinspiration.com/cgi-bin/properties, Molinspiration Cheminformatics. A principal component analysis (PCA) program was developed in-house in a bi-dimensional space regarding the above mentioned variables. The *O. basilicum* L. essential oil components were encoded, according to the terpenoid classification, as monoterpenoid hydrocarbons (M), oxygenated monoterpenes (OM), sesquiterpenoid hydrocarbons (S), and oxygenated sesquiterpenes (OS). Furthermore, the number of cyclic moieties in the terpenoid structures were also considered (e.g., 1 – for monocyclic structure, 2 – for bicyclic, etc.). The PCA analysis was performed by using the centered data and cross validation method.

## Supporting Information

File 1 Complexation data, principal component analysis data, GC–MS analysis chromatograms and mass spectra for *O. basilicum* L. essential oil (raw, degraded or recovered from the β-CD complexes).
