# Supplementary material for: Thermal and oxidative stability of the Ocimum basilicum L. essential oil/β-cyclodextrin supramolecular system
Source: Beilstein J Org Chem. 2014 Nov 28;10:2809–20. doi: 10.3762/bjoc.10.298 (PMC4273302; doi:10.3762/bjoc.10.298)
Supplement: File 1 — Complexation data, principal component analysis data, GC–MS analysis chromatograms and mass spectra for O. basilicum L. essential oil (raw, degraded or recovered from the β-CD complexes). [file Beilstein_J_Org_Chem-10-2809-s001.pdf]

Supporting Information

for

Thermal and oxidative stability of the *Ocimum basilicum* L. essential oil/β-cyclodextrin supramolecular system

Daniel I. Hădărugă[1*], Nicoleta G. Hădărugă[2], Corina I. Costescu[2], Ioan David[2], Alexandra T. Gruia[3]

Address: [1]Department of Applied Chemistry, Organic and Natural Compounds Engineering, *Polytechnic* University of Timişoara, Carol Telbisz 6, 300001, Timişoara, Romania, [2]Department of Food Science, Banat's University of Agricultural Sciences and Veterinary Medicine "King Michael I of Romania" – Timişoara, Calea Aradului 119, 300645, Timişoara, Romania and [3]Regional Centre for Immunology and Transplant, County Clinical Emergency Hospital Timişoara, Iosif Bulbuca Blvd. 10, 300736, Timişoara, Romania

Email: Daniel I. Hădărugă - daniel.hadaruga@upt.ro

[*]Corresponding author

Complexation data, principal component analysis data, GC–MS analysis chromatograms and mass spectra for *O. basilicum* L. essential oil (raw, degraded or recovered from the βCD complexes)

Complexation data

Table 1: *Ocimum basilicum* L. essential oil/β-cyclodextrin nanoencapsulation process

N°	*Essential oil*	*Cyclodextrin*	$M_{main\ compds.}$ (g/mol)[a]	*Conc. of ess. oil solution* (mg/mL)	$m_{ess.oil}$ (mg)	m_{CD} (mg)	$m_{complex}$ (mg)	*Yield* (%)
1	Basil (*Ocimum basilicum* L.)	β-Cyclodextrin	148/154	15.3	77.4	670.9	555.1	74.18

[a] The main compounds were methyl chavicol (estragole) and linalool

Table 2: Codes and degradation conditions for the *O. basilicum* L. essential oil and its β-cyclodextrin complex

N°	*Sample*	*Codes*	m_{sample} (mg)	*Degr. temp.* (°C)	*Degr. time* (min.)
1	*Ocimum basilicum* L. essential oil	*B*	50	-	120
2		B_{50}		50	
3		B_{100}		100	
4		B_{150}		150	
5	*Ocimum basilicum* L. essential oil/β-cyclodextrin complex	*B/βCD*	450	-	120
6		$B/\beta CD_{50}$		50	
7		$B/\beta CD_{100}$		100	
8		$B/\beta CD_{150}$		150	

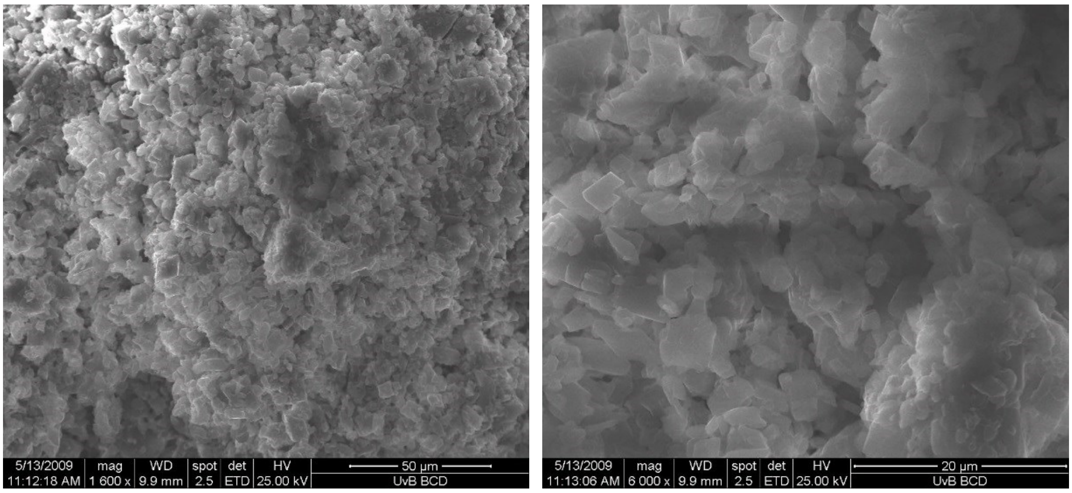

Figure 1: SEM images for the *Ocimum basilicum* L. essential oil/β-cyclodextrin complex

PCA analysis

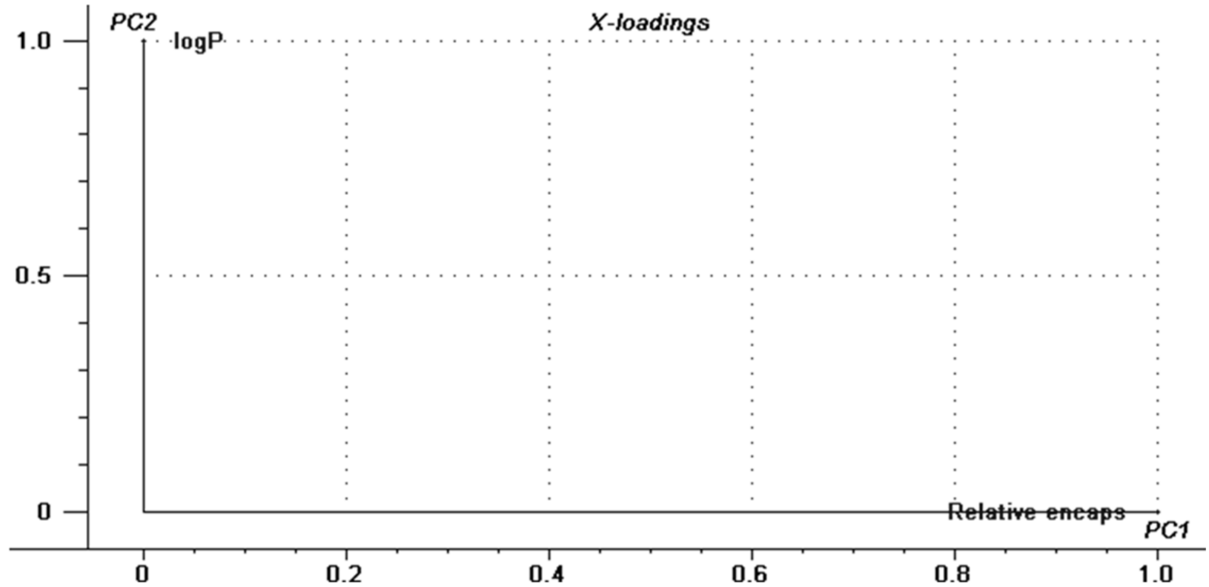

Figure 2: The loadings plot from the PCA analysis of the *O. basilicum* L. essential oil compounds nanoencapsulation in β-cyclodextrin (the encapsulation efficiency – "Relative encapsualtion" and hydrophobicity – "logP", were the PCA variables)

Table 3: Scores data from the PCA analysis of *O. basilicum* essential oil compounds nanoencapsulation in βCD

	PC_01 (Samples)	PC_02 (Samples)
M1	10.078	-0.533
OM2	10.562	-1.432
OM1	-50.197	-2.210
OM	83.144	-0.929
OM2	-4.551	-1.989
OM1	45.112	-1.327
OM1	6.414	-1.635
S1	19.144	1.222
S2	11.483	1.091
S2	16.619	1.027
S2	13.029	0.742
S1	3.953	1.157
S3	3.736	1.461
S2	13.387	0.923
S2	10.888	1.607
OS3	-4.255	-0.240
OS3	-12.409	-1.356e-02
OS2	-54.093	0.821
OS4	-21.403	0.121
OS4	-69.552	0.113
OS4	-31.090	2.402e-02

Table 4: Loadings data from the PCA analysis of *O. basilicum* essential oil compounds nanoencapsulation in βCD

	PC_01 (X-Variables)	PC_02 (X-Variables)
Relative encapsulation (%)	1.000	7.580e-05
logP	-7.595e-05	1.000

GC-MS analysis

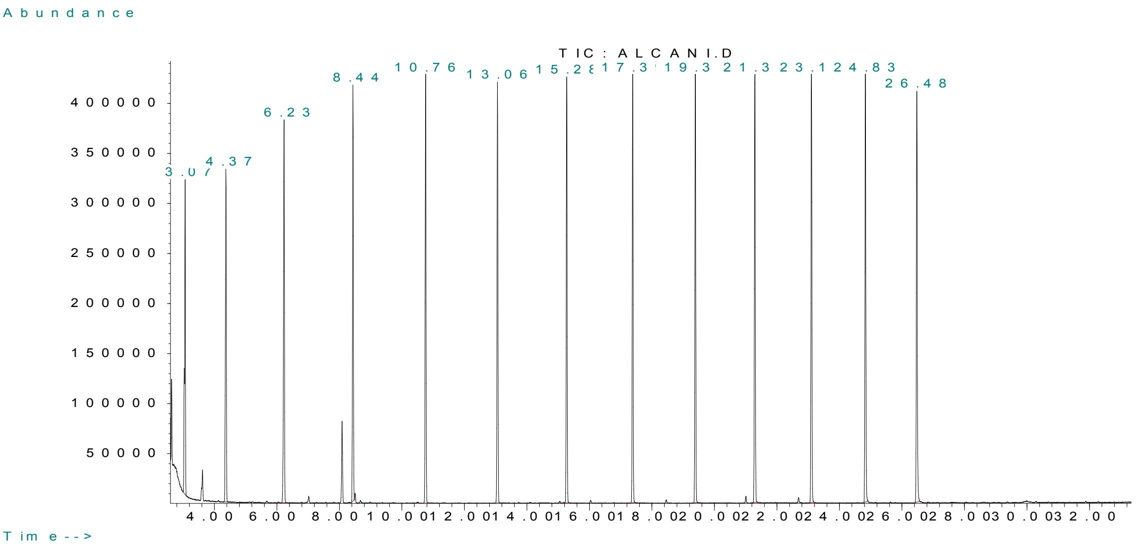

Figure 3: Gas chromatogram from the GC-MS analysis of C_8-C_{20} alkane standard solution

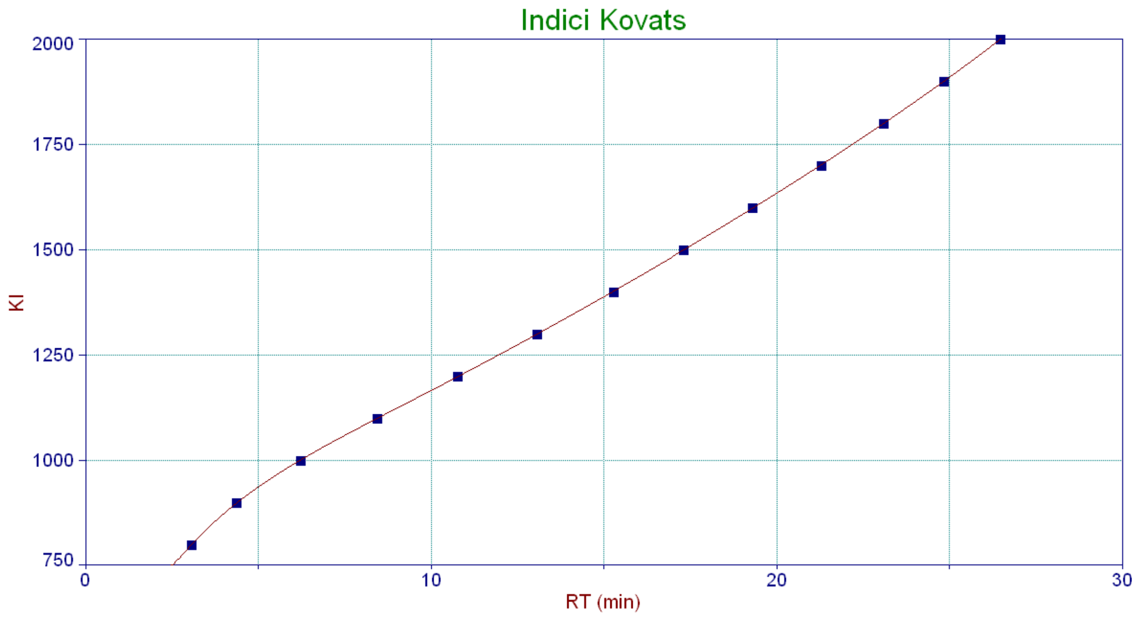

Figure 4: *Kovats index vs. Retention time* correlation for the GC-MS analysis of alkane standard solution (used for determination of *KIs* of *Ocimum basilicum* L. essential oil components)

Table 5: The main compounds identified in raw and degraded *O. basilicum* L. essential oils (classes of compounds and their chemical structures). The number of compound corresponds to Table 1 (from the main article)

N°	Compound name	Compound structure
Monoterpenoid hydrocarbons, monocyclic (M1)		
1	Limonene	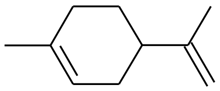
Oxygenated monoterpenoids, acyclic (OM)		
4	Linalool	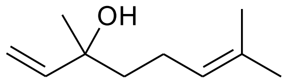
Oxygenated monoterpenoids, monocyclic (OM1)		
3	Linalool oxide	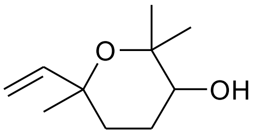
7	Carvone	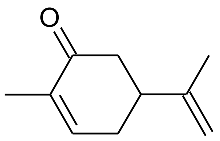
Oxygenated monoterpenoids, bicyclic (OM2)		
2	Eucalyptol	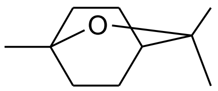
5	Camphor	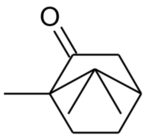
Phenolic derivatives		
6	Methyl chavicol	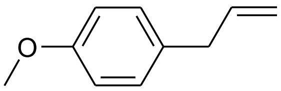
Sesquiterpenoid hydrocarbons, monocyclic (S1)		
8	β-Elemen	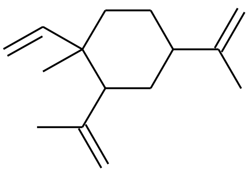
12	Humulene	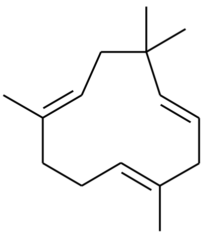

Sesquiterpenoid hydrocarbons, bicyclic (S2)

9	α-Bergamotene	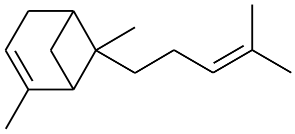
10	β-Caryophyllene	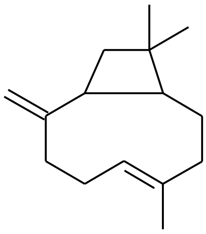
11	α-Guaiene	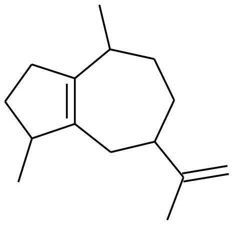
14	α-Bulnesene	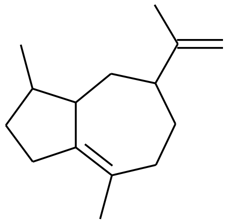
15	γ-Cadinene	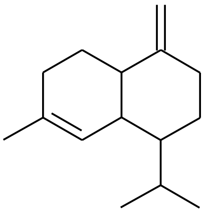

Sesquiterpenoid hydrocarbons, tricyclic (S3)

13	β-Cubebene	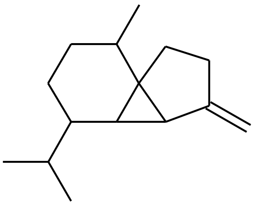

Oxygenated sesquiterpenoid hydrocarbons, bicyclic (OS2)

18	α-Cadinol	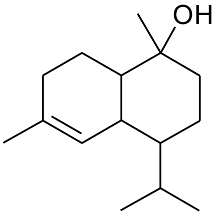

Oxygenated sesquiterpenoid hydrocarbons, tricyclic (OS3)		
16	Spathulenol	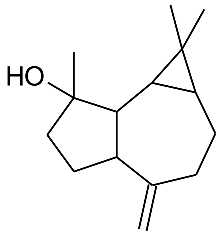
17	Caryophyllene oxide	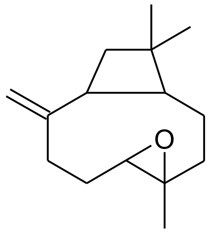
Oxygenated sesquiterpenoid hydrocarbons, tetracyclic (OS4)		
21	Aristolene epoxide	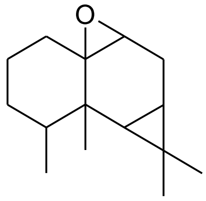
23	Aromadendrene epoxide	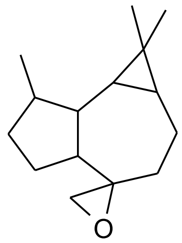

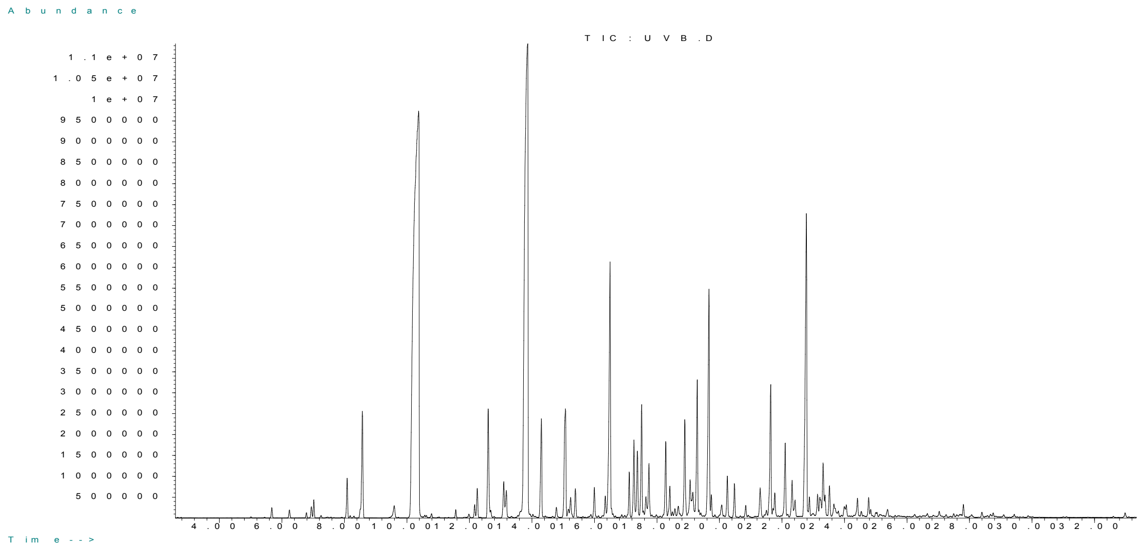

Figure 5: The GC chromatogram of the raw *O. basilicum* L. essential oil

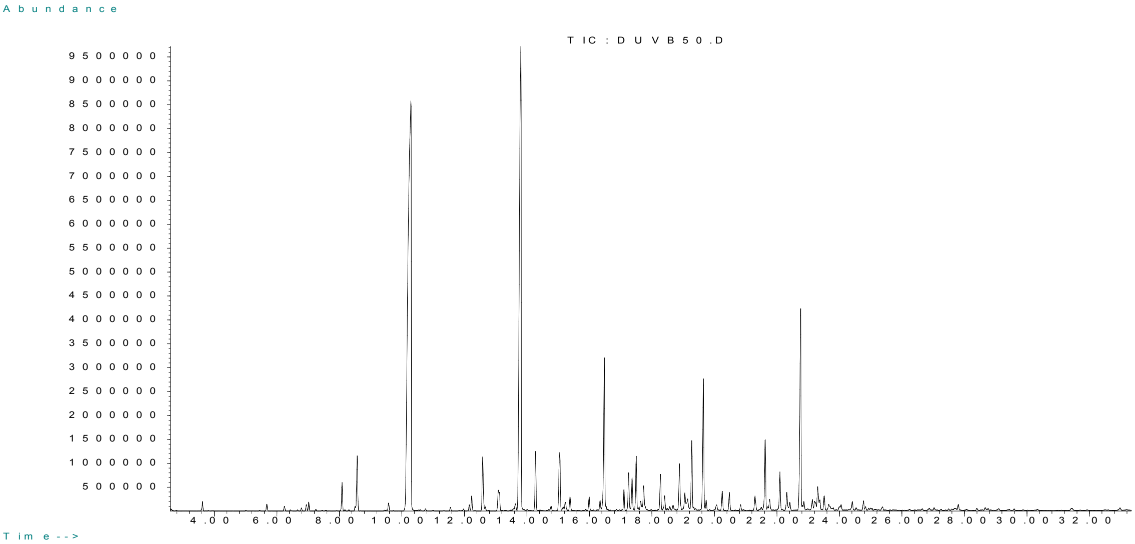

Figure 6: The GC chromatogram of the *O. basilicum* L. essential oil degraded at 50°C

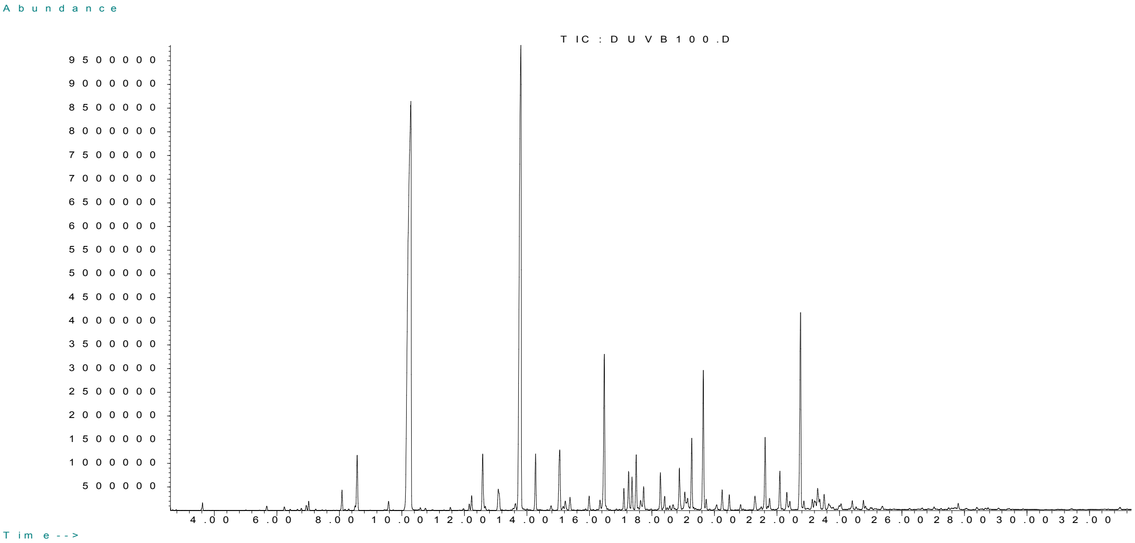

Figure 7: The GC chromatogram of the *O. basilicum* L. essential oil degraded at 100°C

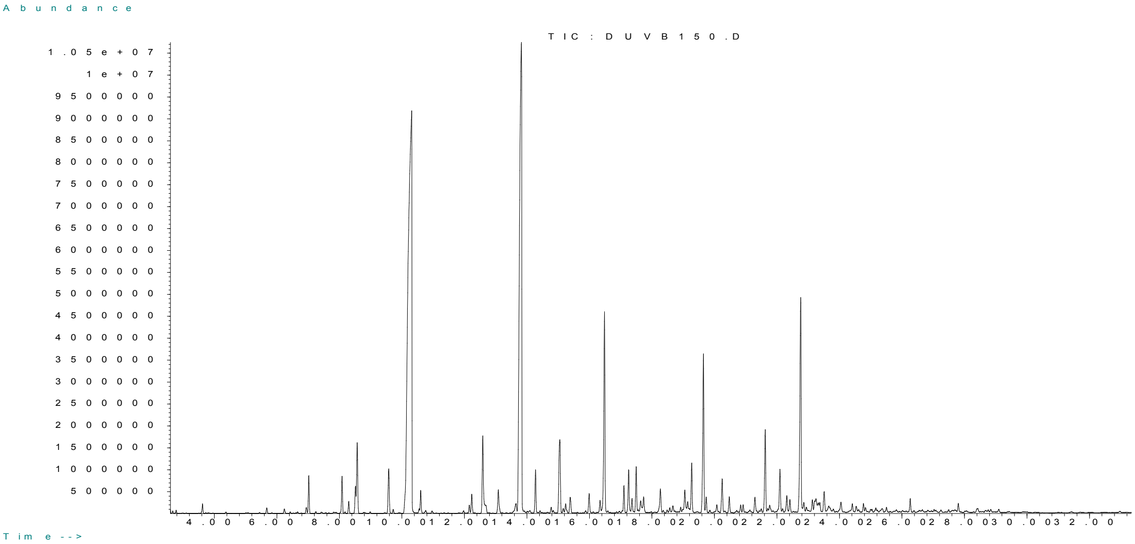

Figure 8: The GC chromatogram of the *O. basilicum* L. essential oil degraded at 150°C

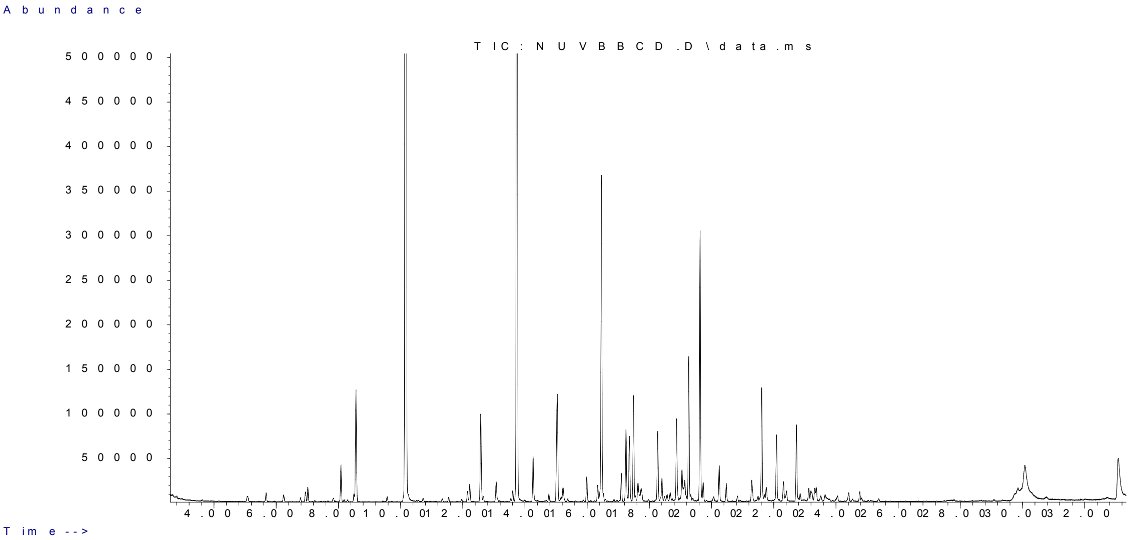

Figure 9: The GC chromatogram of the recovered *O. basilicum* L. essential oil from the non-degraded β-cyclodextrin complex

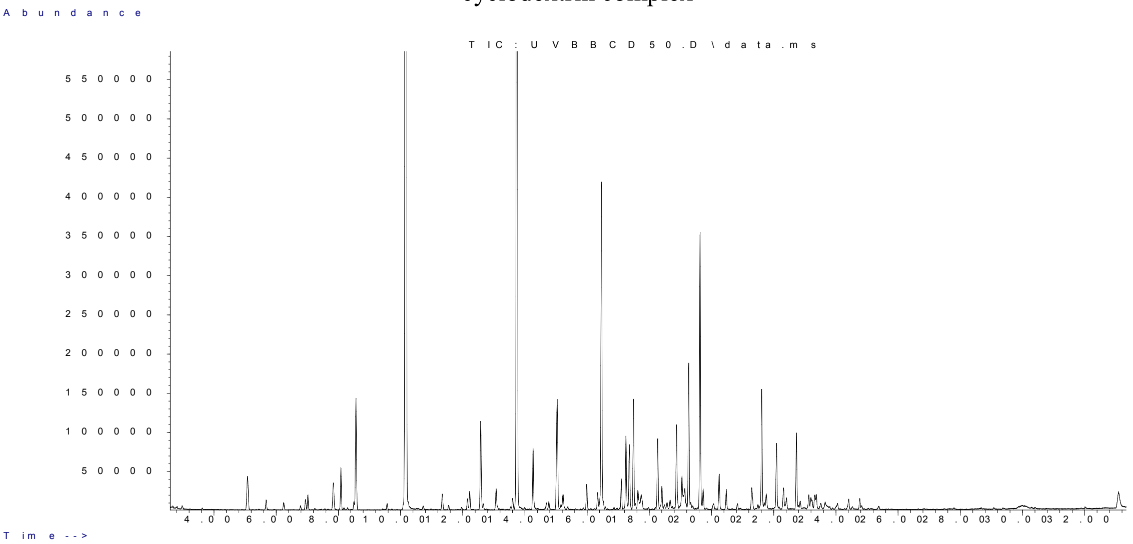

Figure 10: The GC chromatogram of the recovered *O. basilicum* L. essential oil from the degraded β-cyclodextrin complex at 50°C

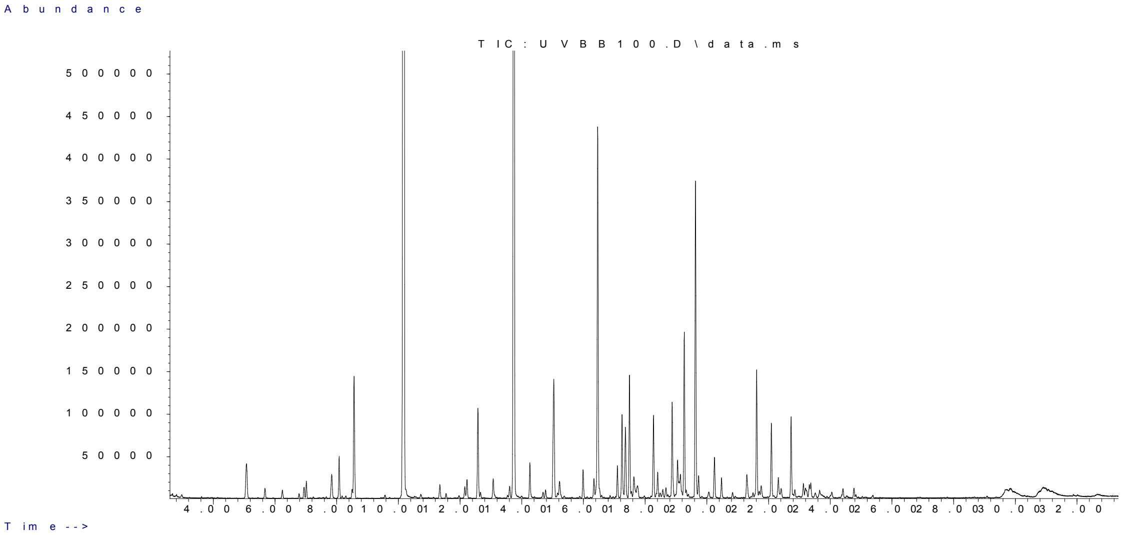

Figure 11: The GC chromatogram of the recovered *O. basilicum* L. essential oil from the degraded β-cyclodextrin complex at 100°C

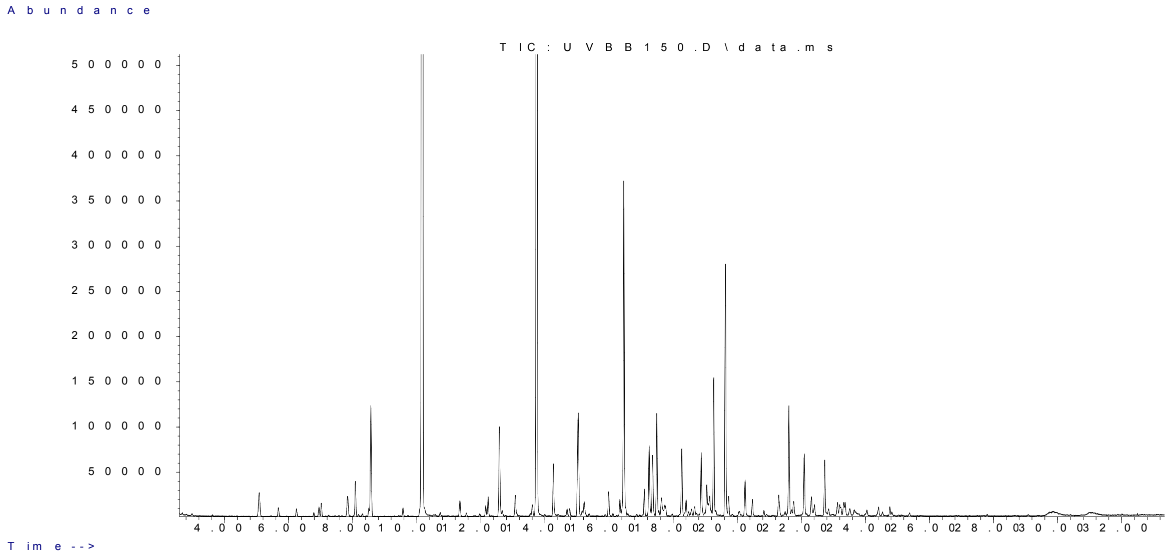

Figure 12: The GC chromatogram of the recovered *O. basilicum* L. essential oil from the degraded β-cyclodextrin complex at 150°C

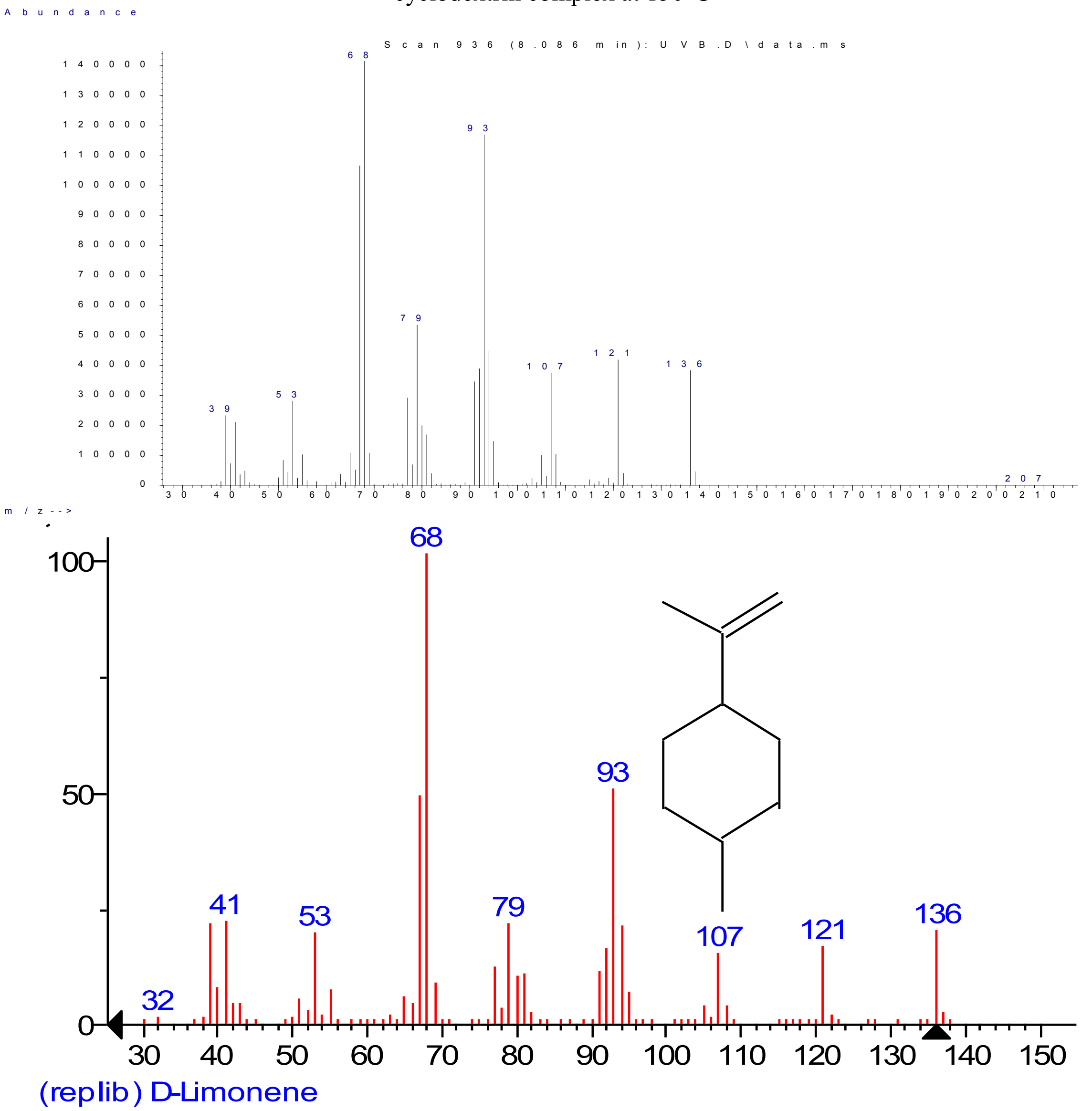

Figure 13: The experimental (up) and from the NIST database (down) MS spectra for limonene identified from the GC-MS analysis of *O. basilicum* L. essential oil

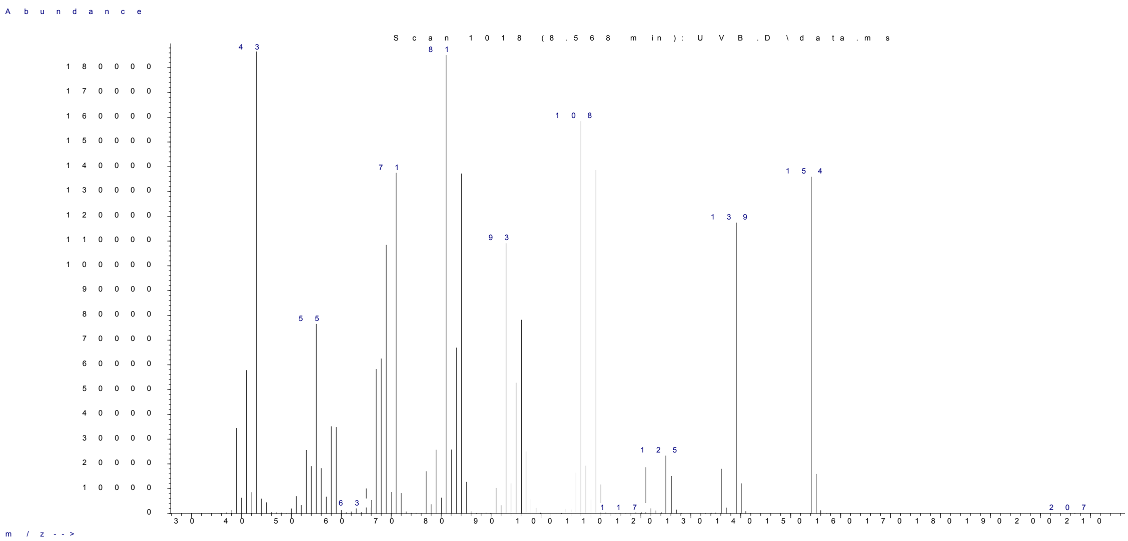

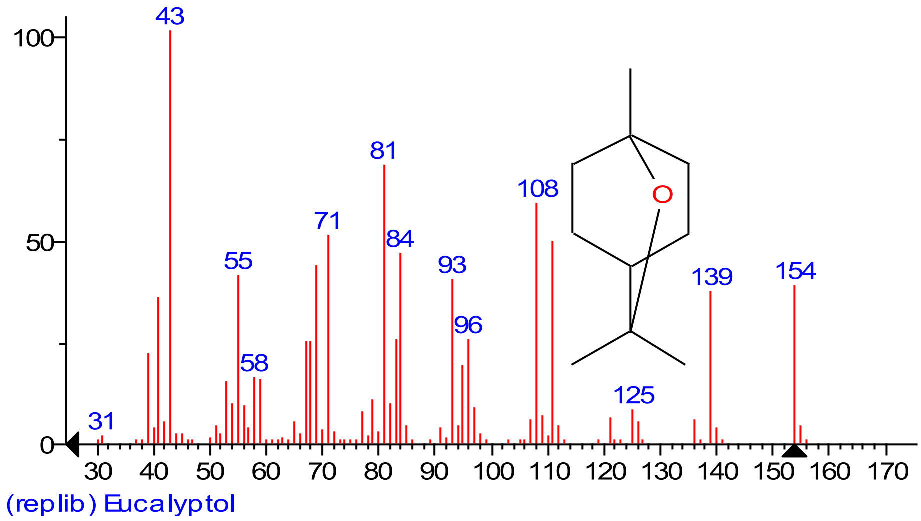

Figure 14: The experimental (up) and from the NIST database (down) MS spectra for eucalyptol identified from the GC-MS analysis of *O. basilicum* L. essential oil

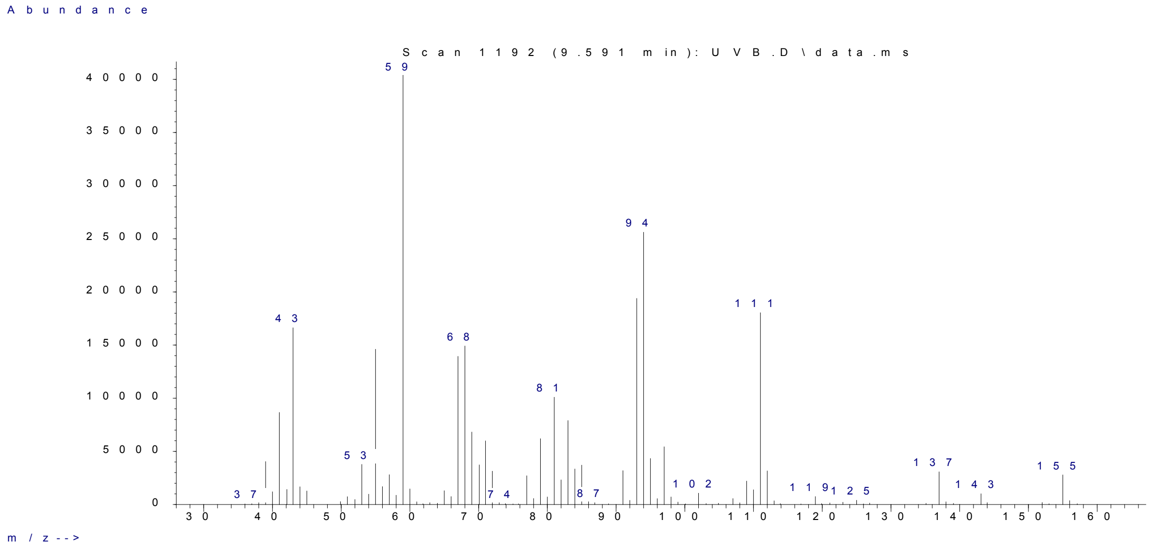

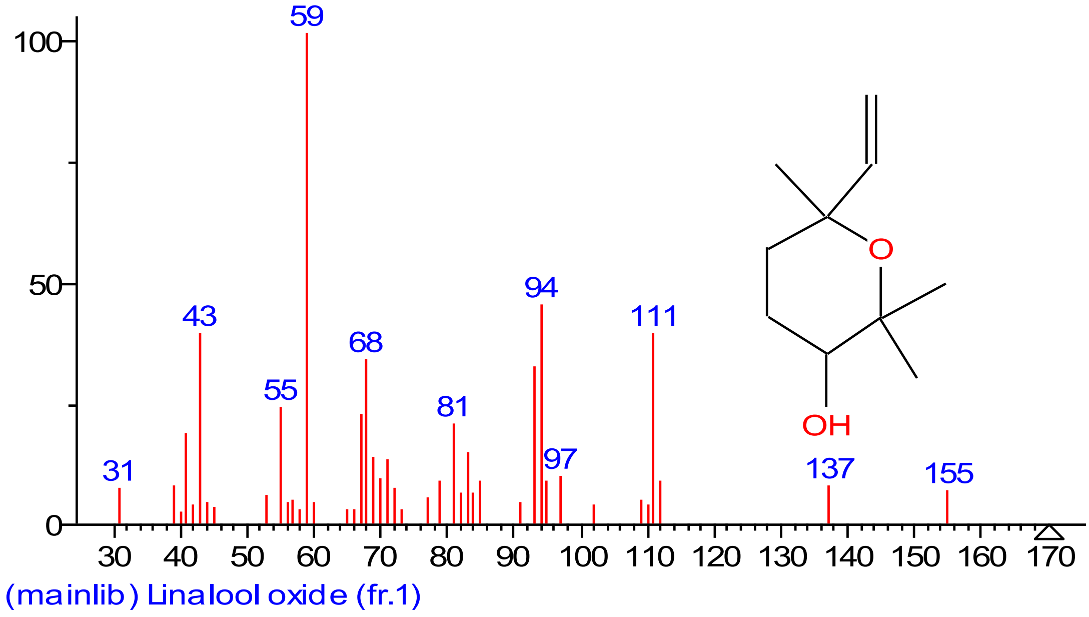

Figure 15: The experimental (up) and from the NIST database (down) MS spectra for linalool oxide identified from the GC-MS analysis of *O. basilicum* L. essential oil

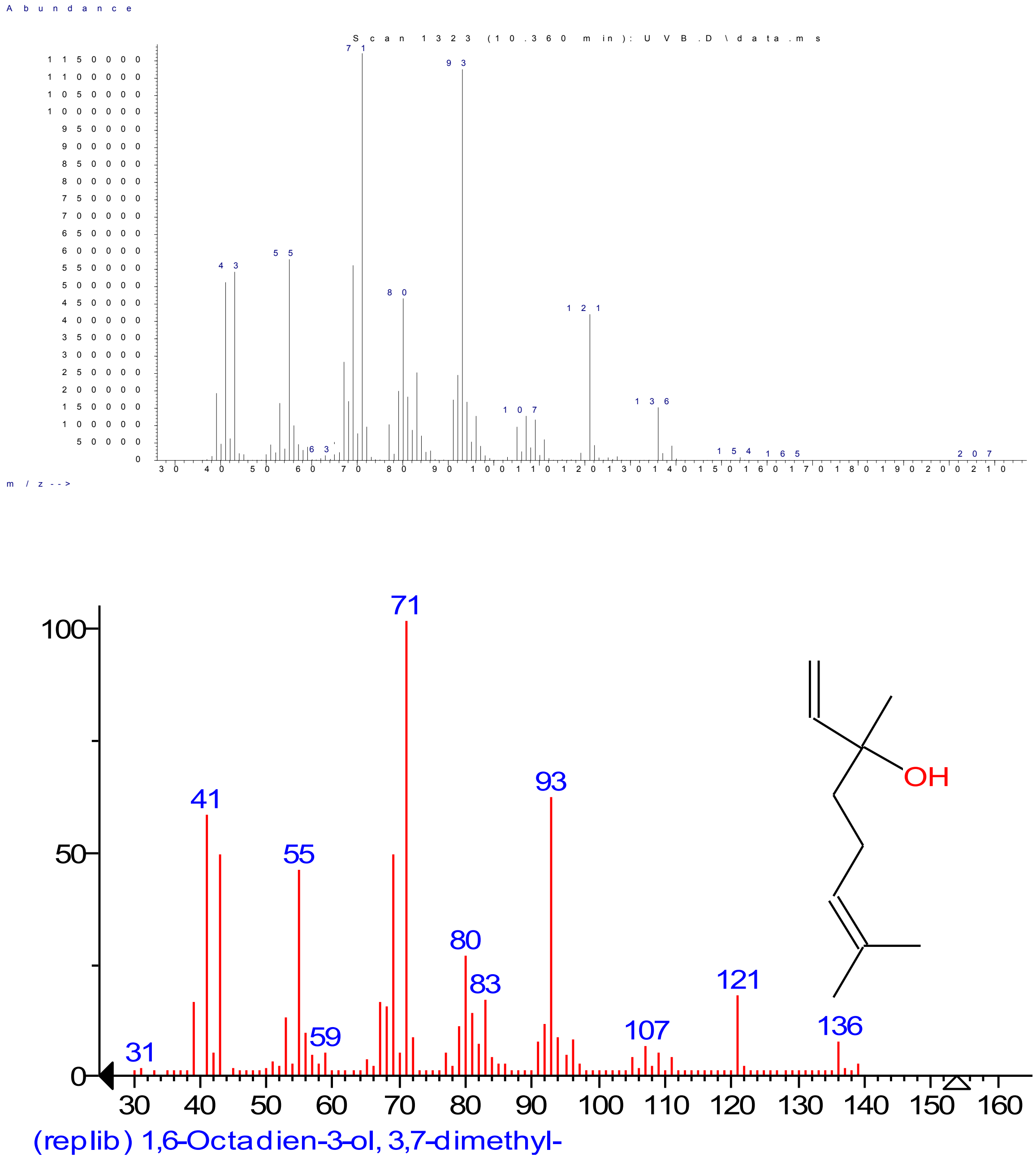

Figure 16: The experimental (up) and from the NIST database (down) MS spectra for linalool identified from the GC-MS analysis of *O. basilicum* L. essential oil

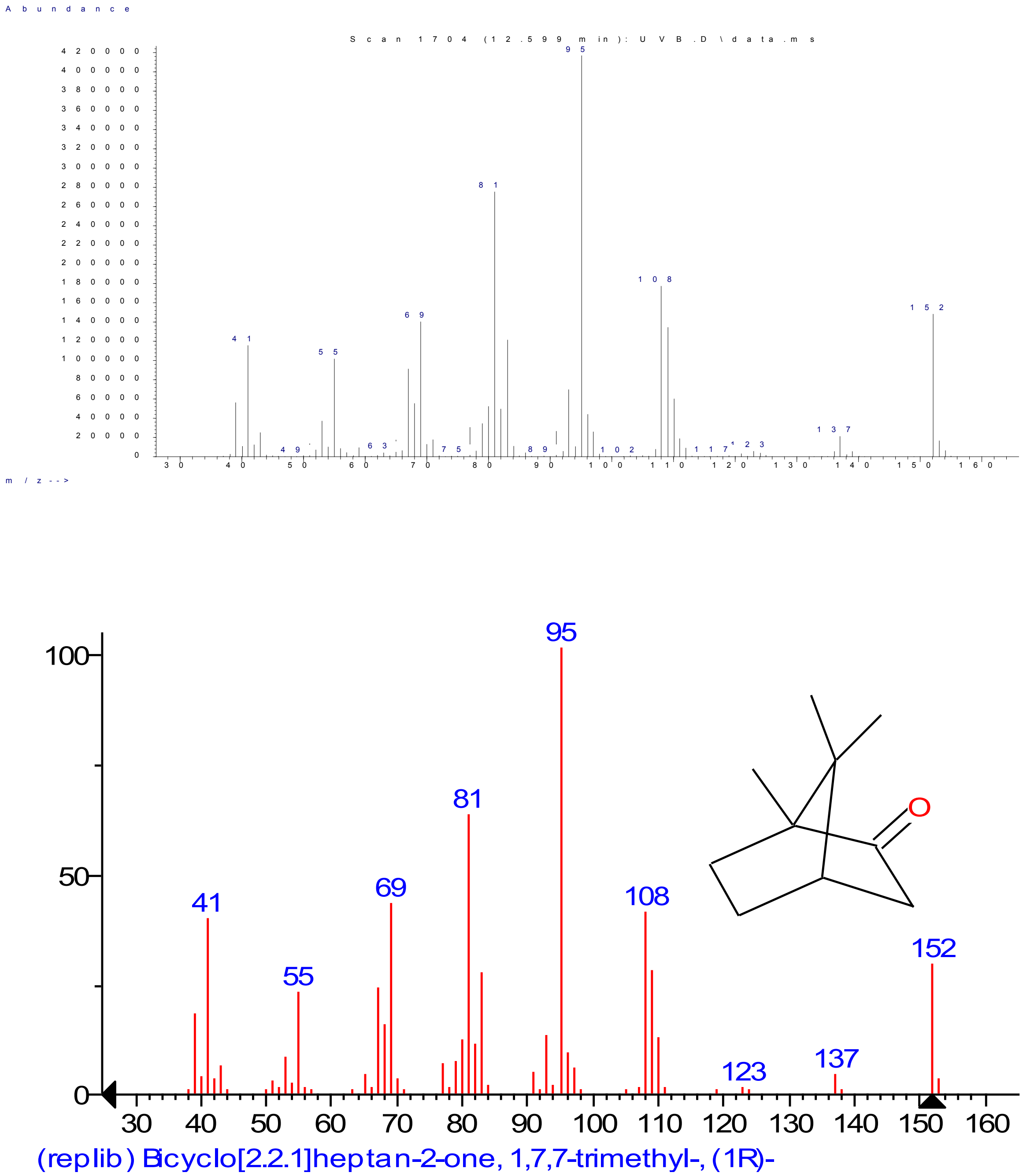

Figure 17: The experimental (up) and from the NIST database (down) MS spectra for camphor identified from the GC-MS analysis of *O. basilicum* L. essential oil

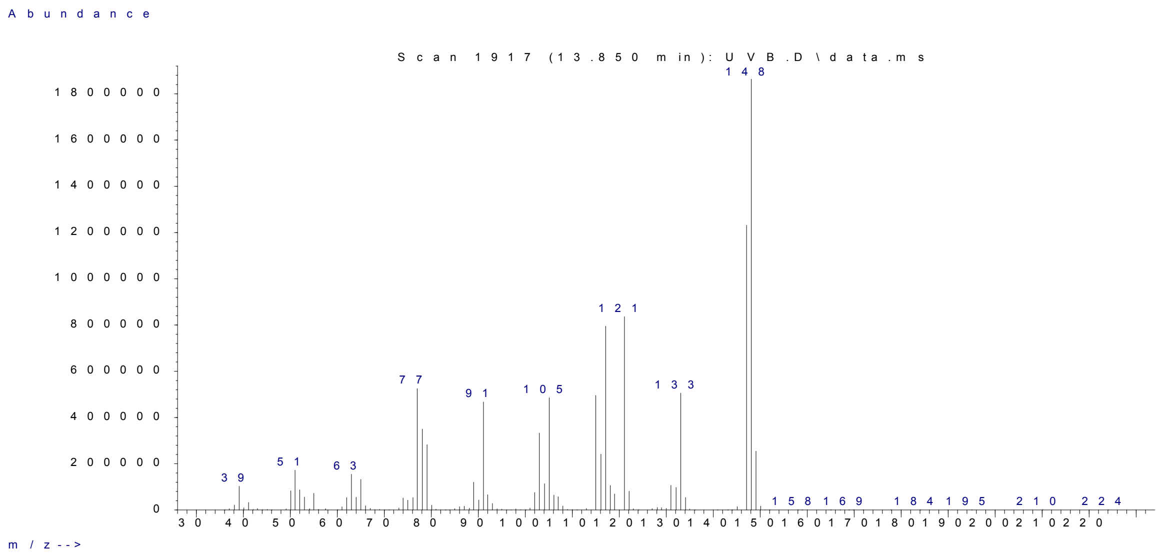

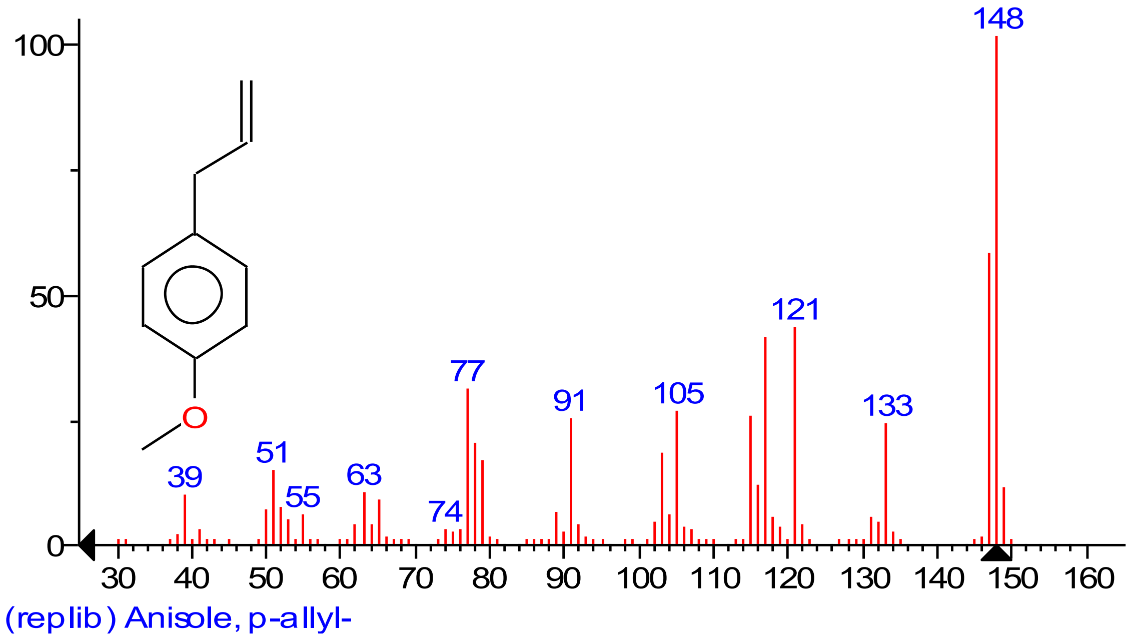

Figure 18: The experimental (up) and from the NIST database (down) MS spectra for methyl chavicol (estragole) identified from the GC-MS analysis of *O. basilicum* L. essential oil

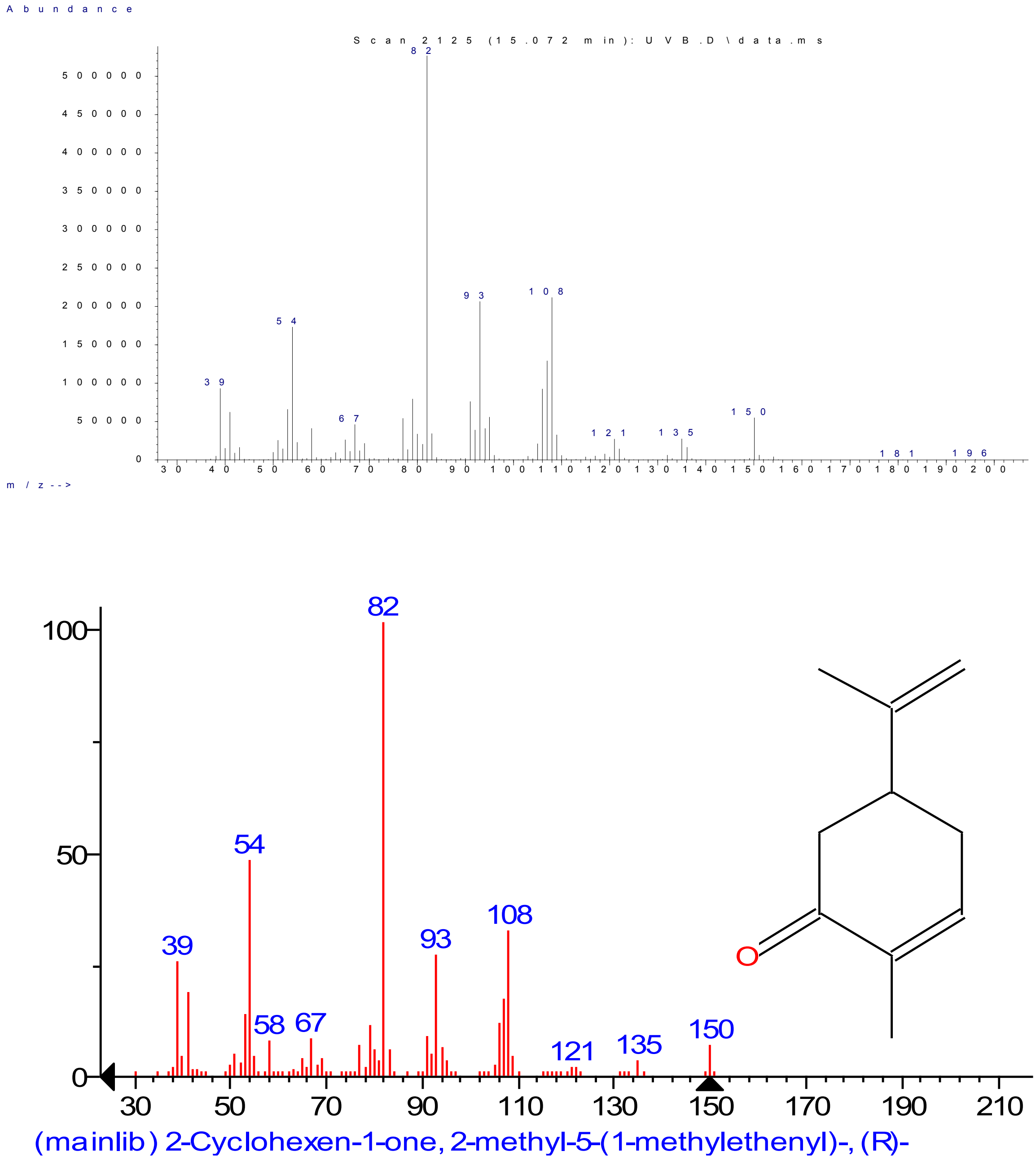

Figure 19: The experimental (up) and from the NIST database (down) MS spectra for carvone identified from the GC-MS analysis of *O. basilicum* L. essential oil

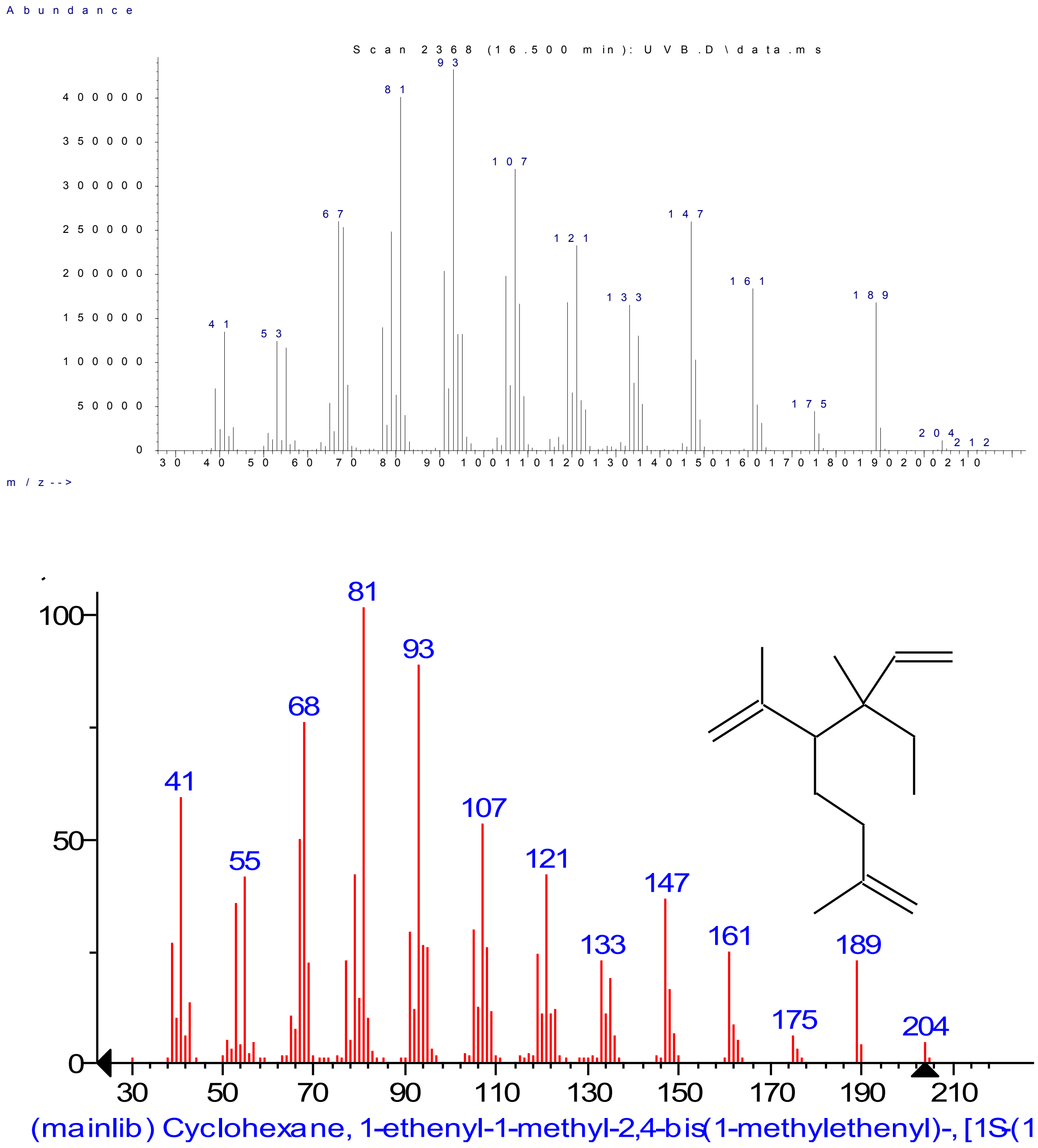

Figure 20: The experimental (up) and from the NIST database (down) MS spectra for β-elemen identified from the GC-MS analysis of *O. basilicum* L. essential oil

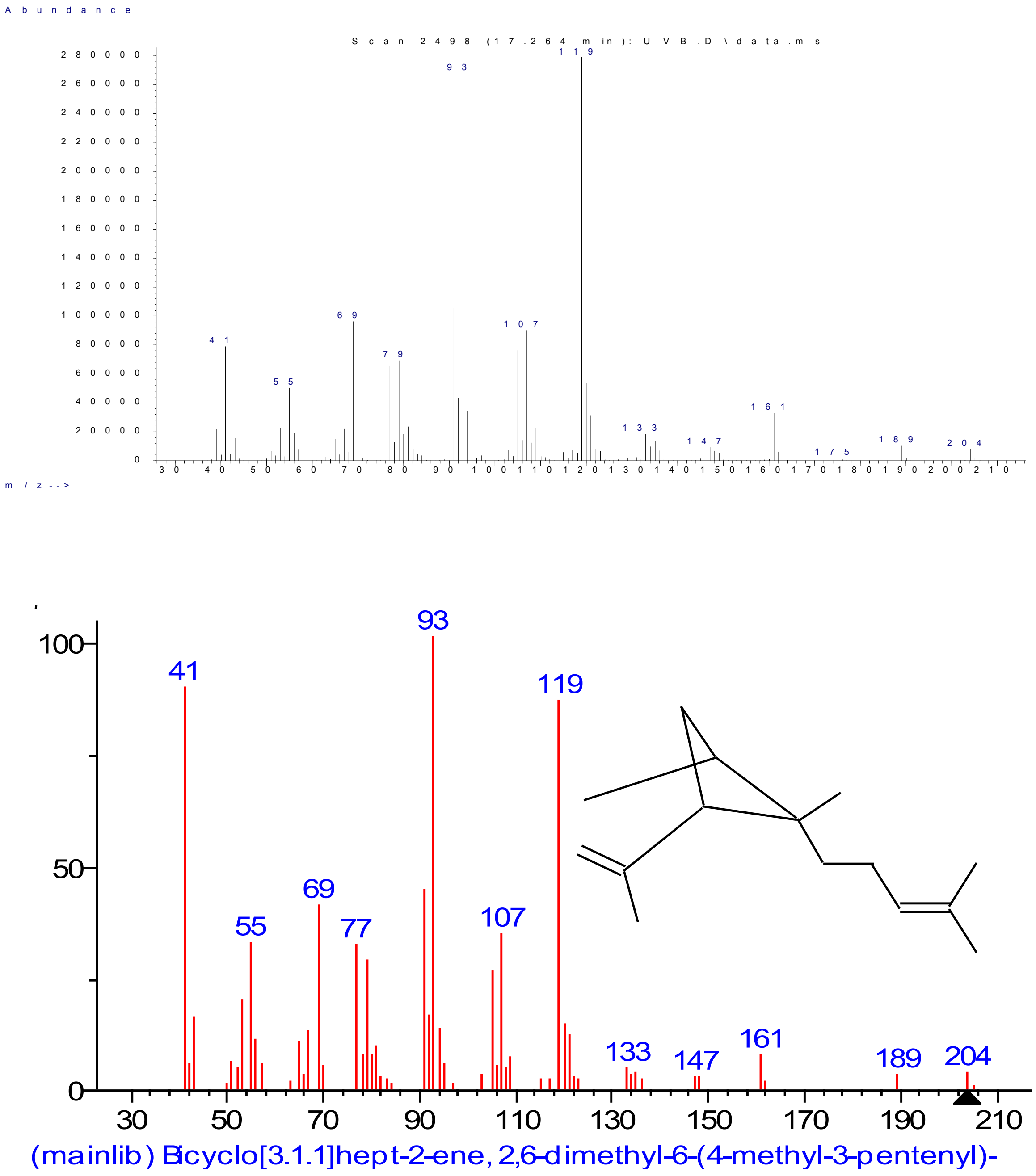

Figure 21: The experimental (up) and from the NIST database (down) MS spectra for α-bergamotene identified from the GC-MS analysis of *O. basilicum* L. essential oil

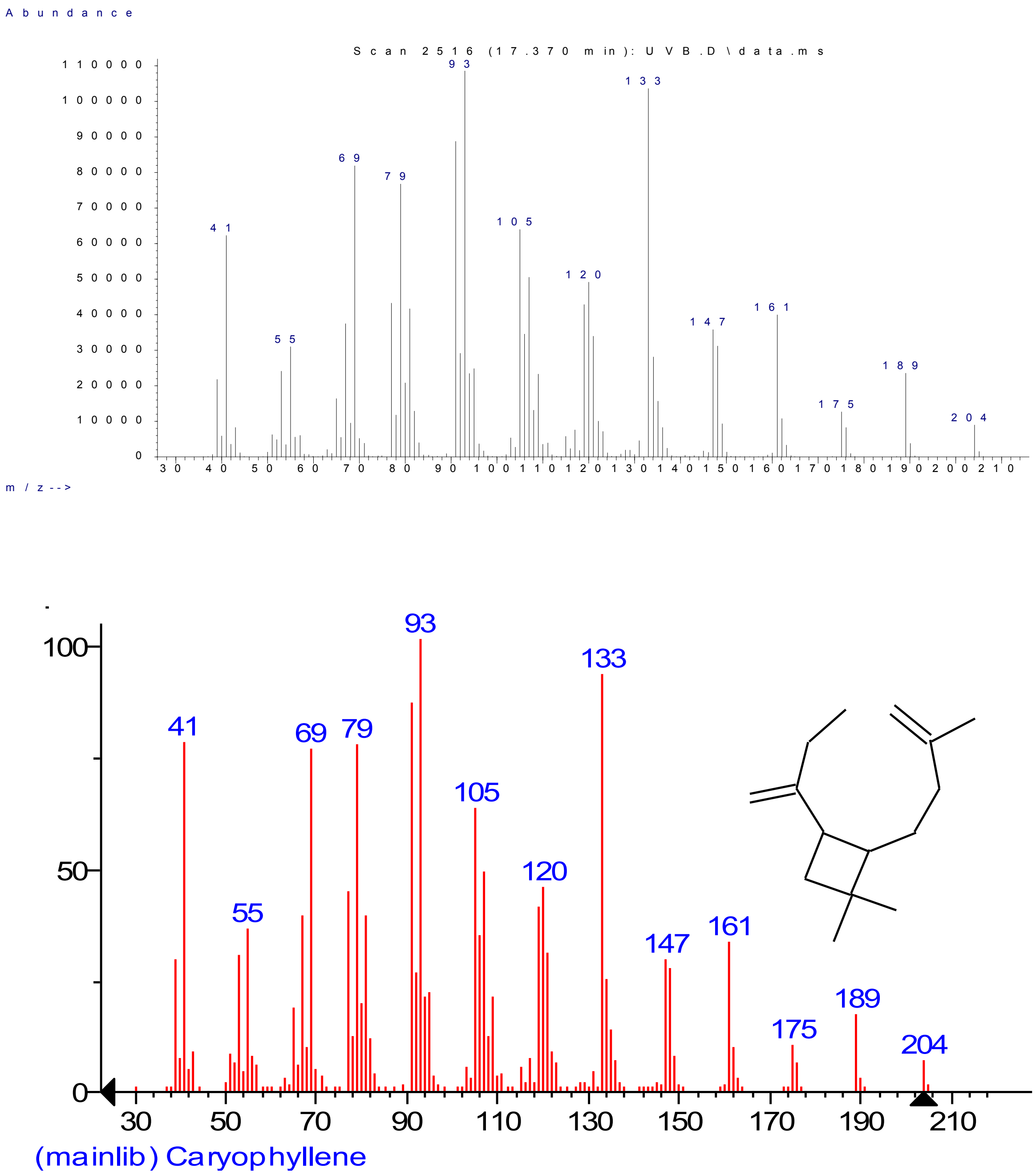

Figure 22: The experimental (up) and from the NIST database (down) MS spectra for β-caryophyllene identified from the GC-MS analysis of *O. basilicum* L. essential oil

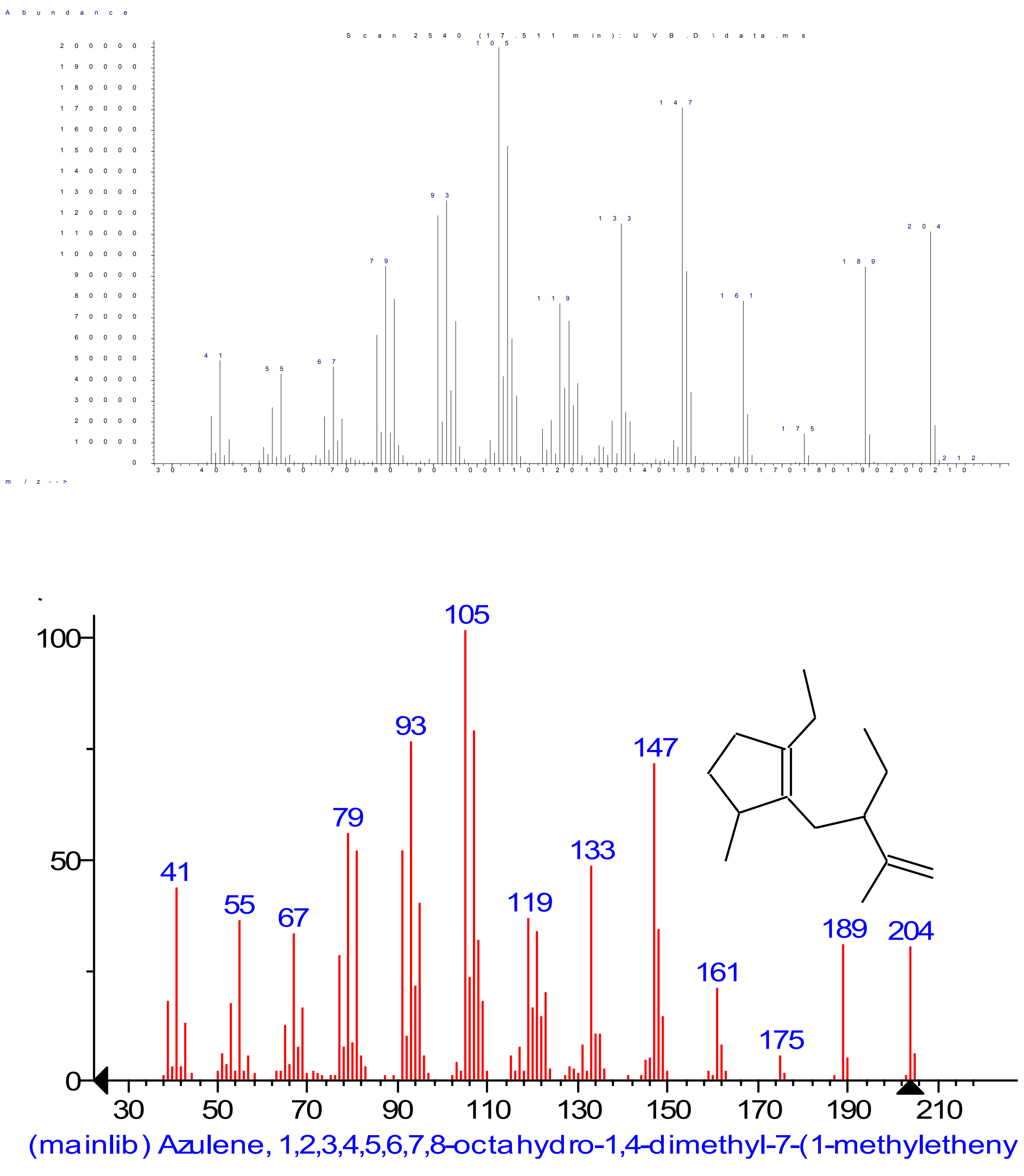

Figure 23: The experimental (up) and from the NIST database (down) MS spectra for α-guaiene identified from the GC-MS analysis of *O. basilicum* L. essential oil

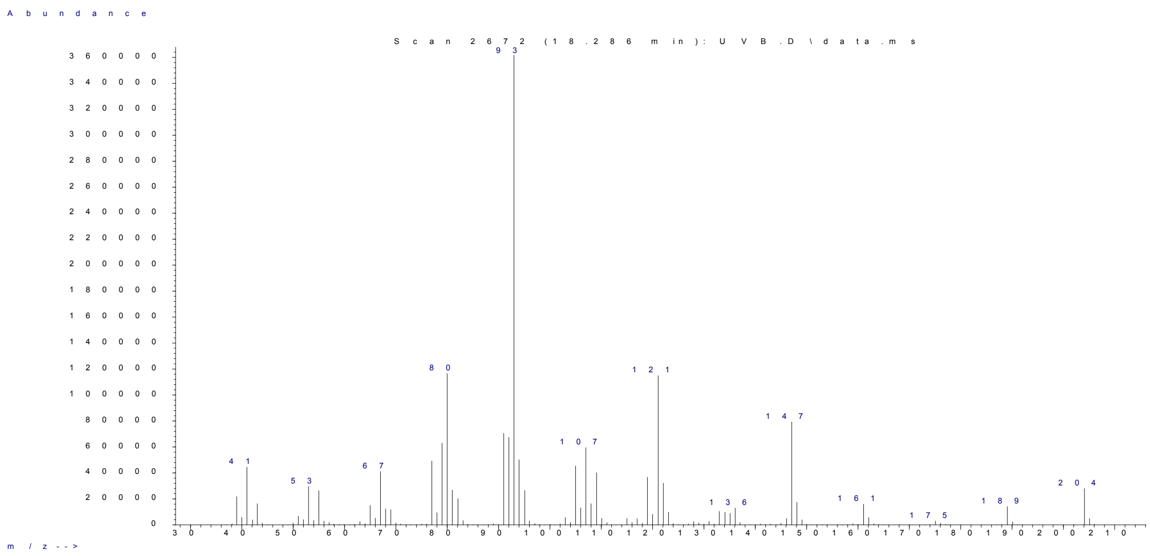

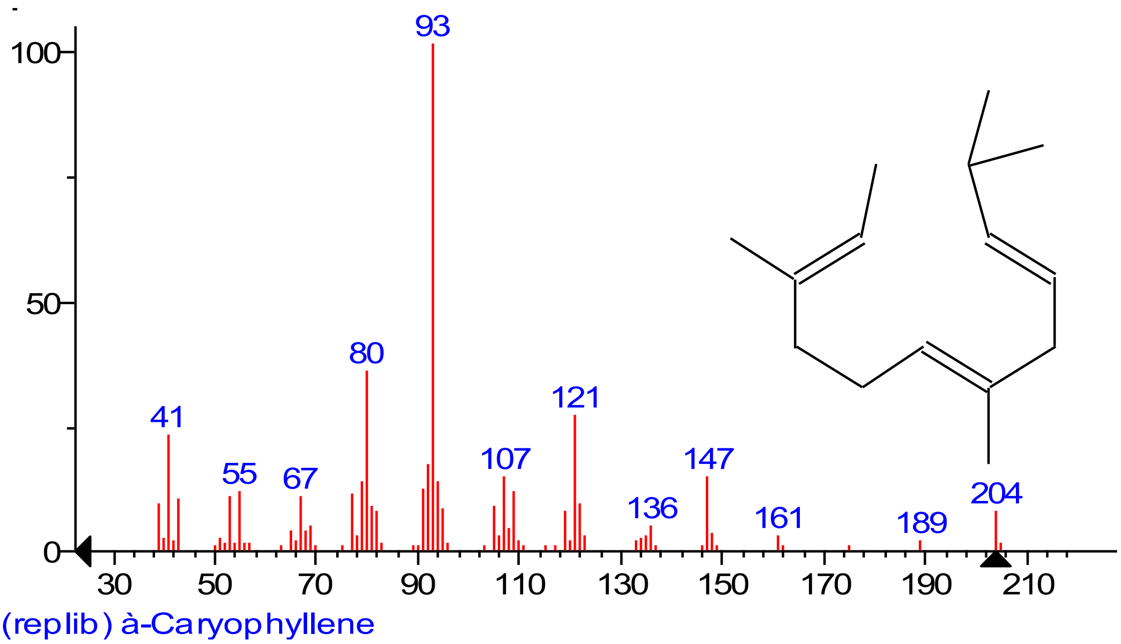

Figure 24: The experimental (up) and from the NIST database (down) MS spectra for humulene identified from the GC-MS analysis of *O. basilicum* L. essential oil

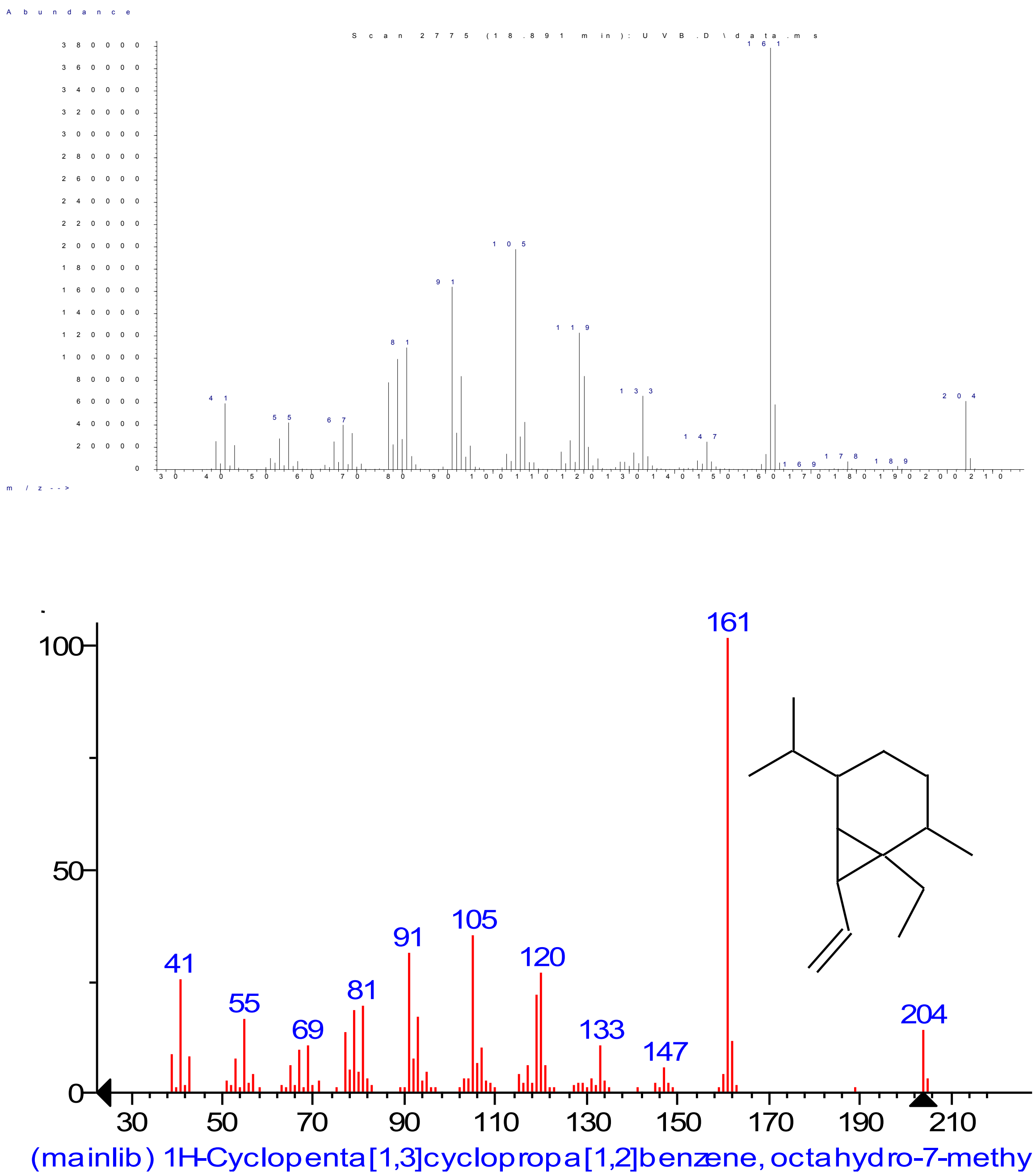

Figure 25: The experimental (up) and from the NIST database (down) MS spectra for β-cubebene identified from the GC-MS analysis of *O. basilicum* L. essential oil

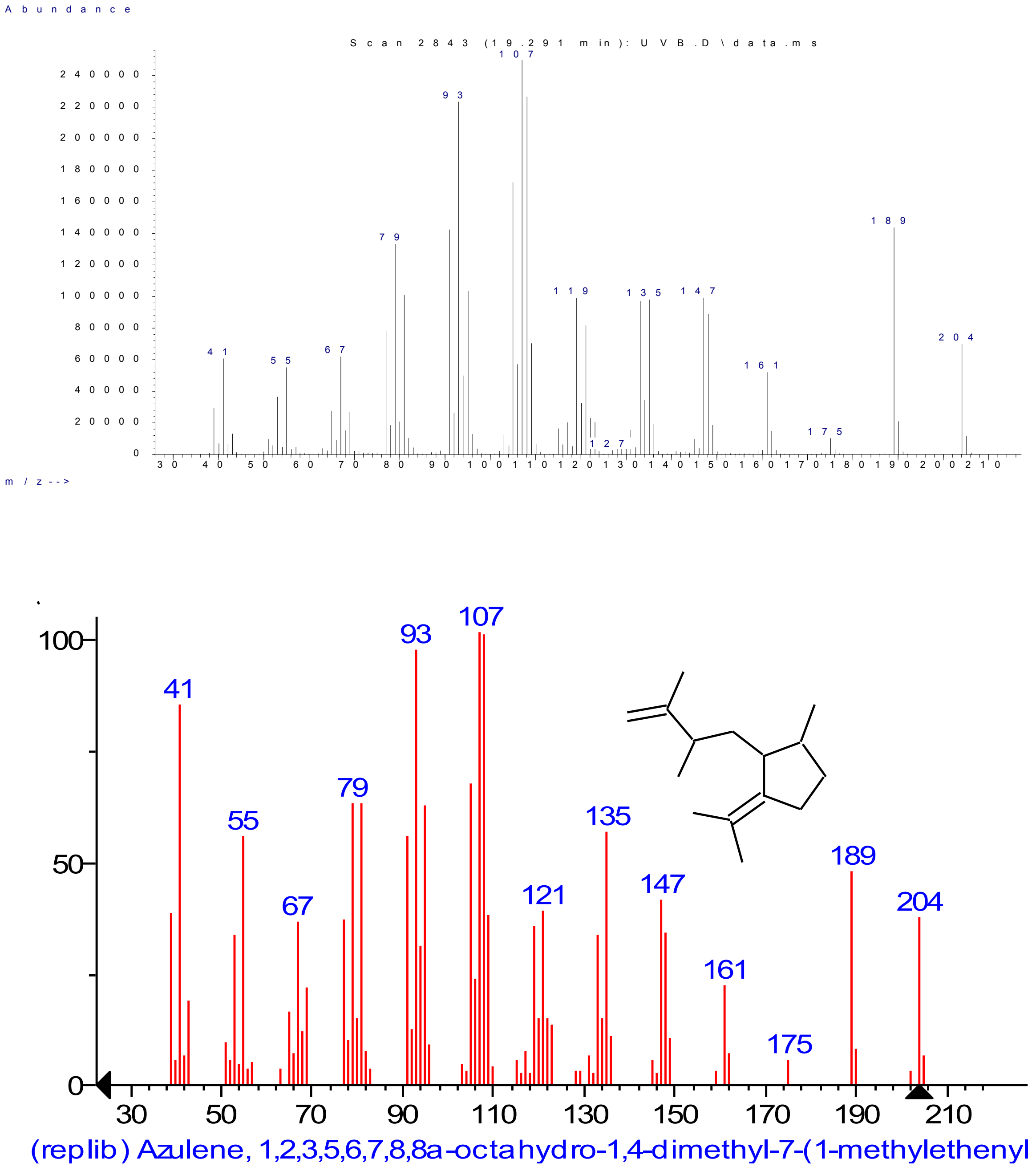

Figure 26: The experimental (up) and from the NIST database (down) MS spectra for α-bulnesene identified from the GC-MS analysis of *O. basilicum* L. essential oil

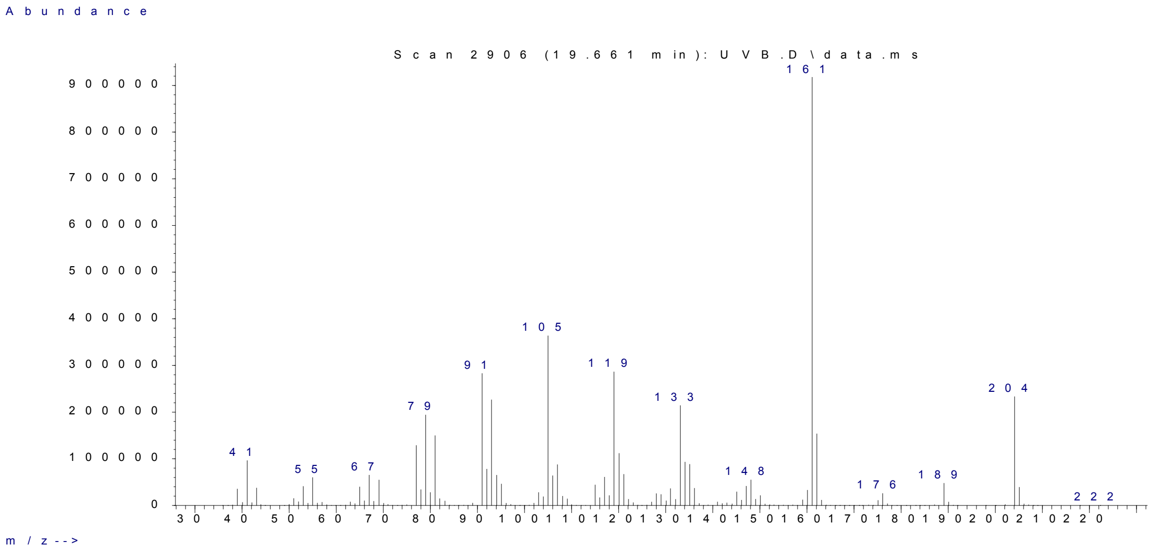

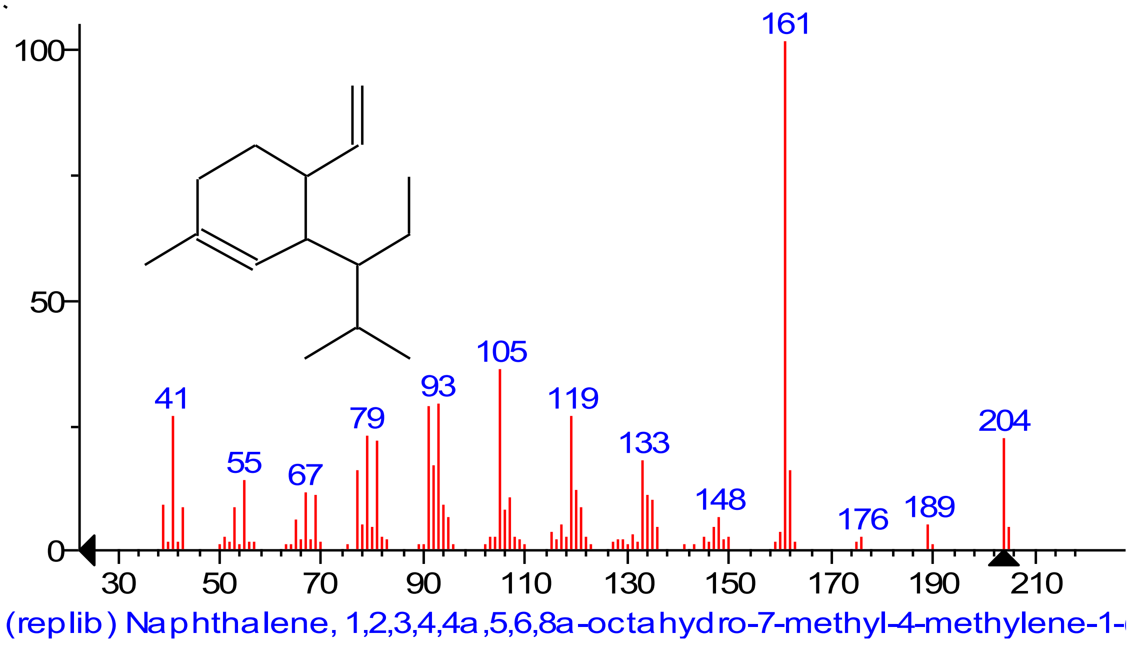

Figure 27: The experimental (up) and from the NIST database (down) MS spectra for γ-cadinene identified from the GC-MS analysis of *O. basilicum* L. essential oil

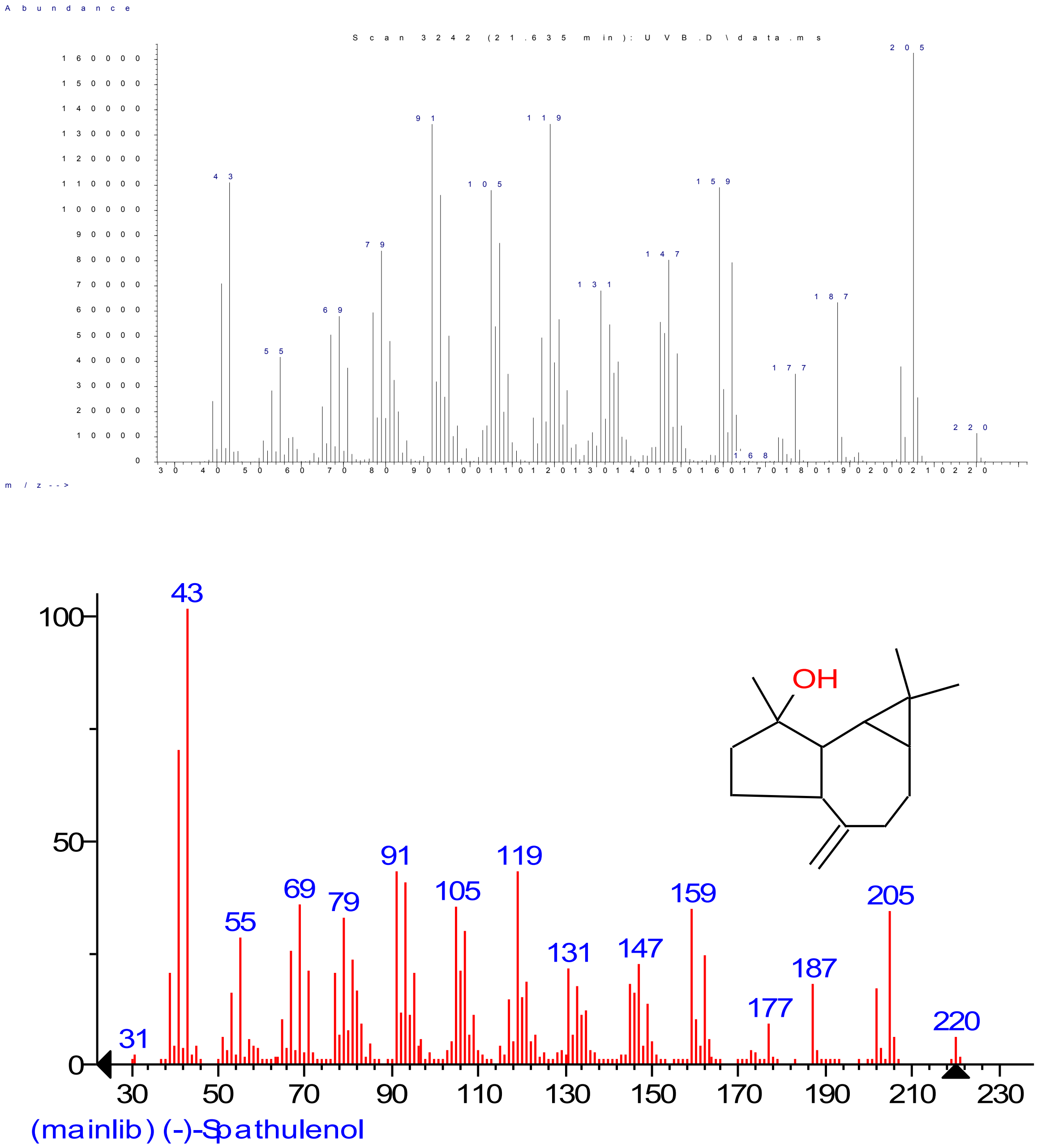

Figure 28: The experimental (up) and from the NIST database (down) MS spectra for spathulenol identified from the GC-MS analysis of *O. basilicum* L. essential oil

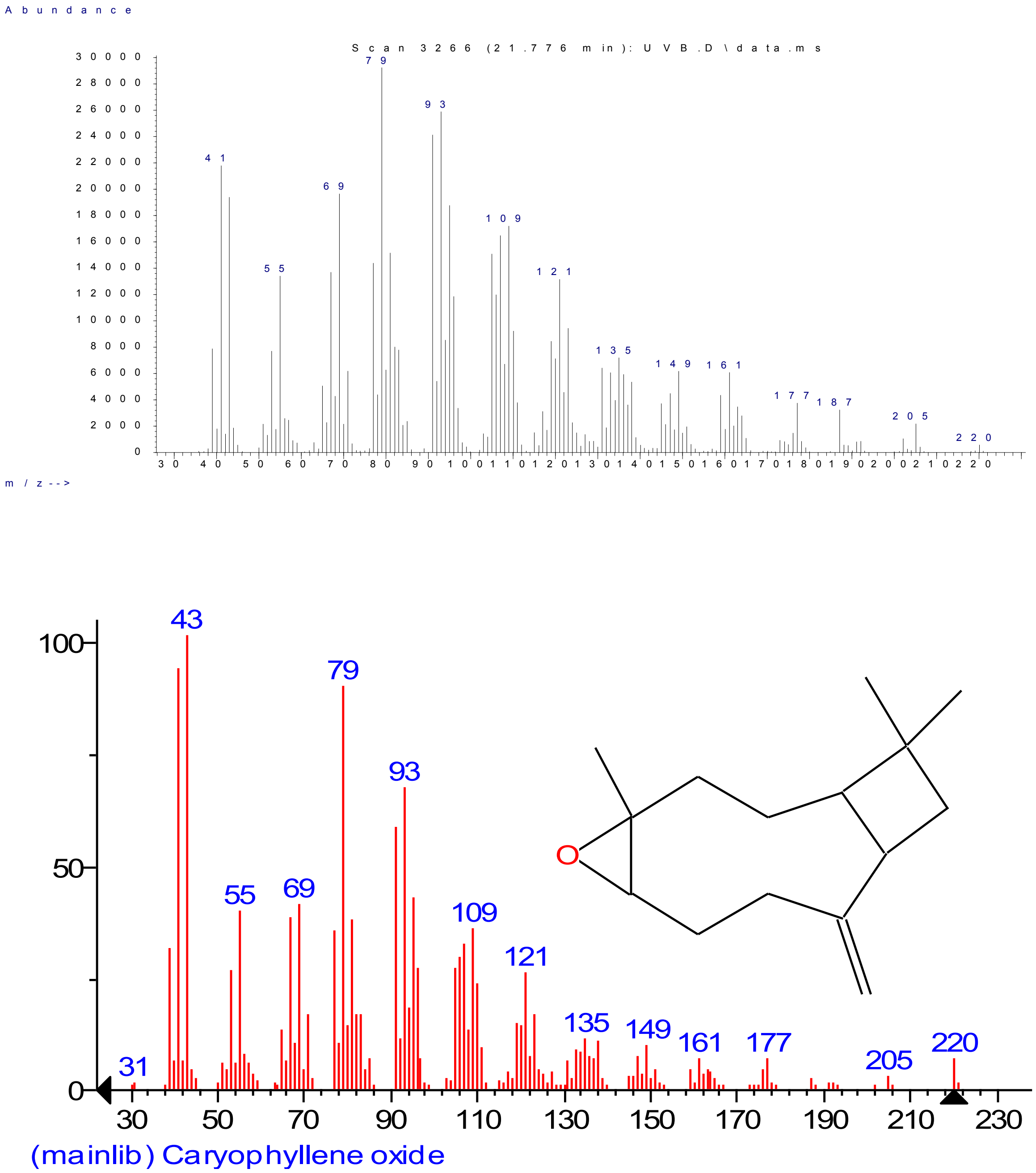

Figure 29: The experimental (up) and from the NIST database (down) MS spectra for caryophyllene oxide identified from the GC-MS analysis of *O. basilicum* L. essential oil

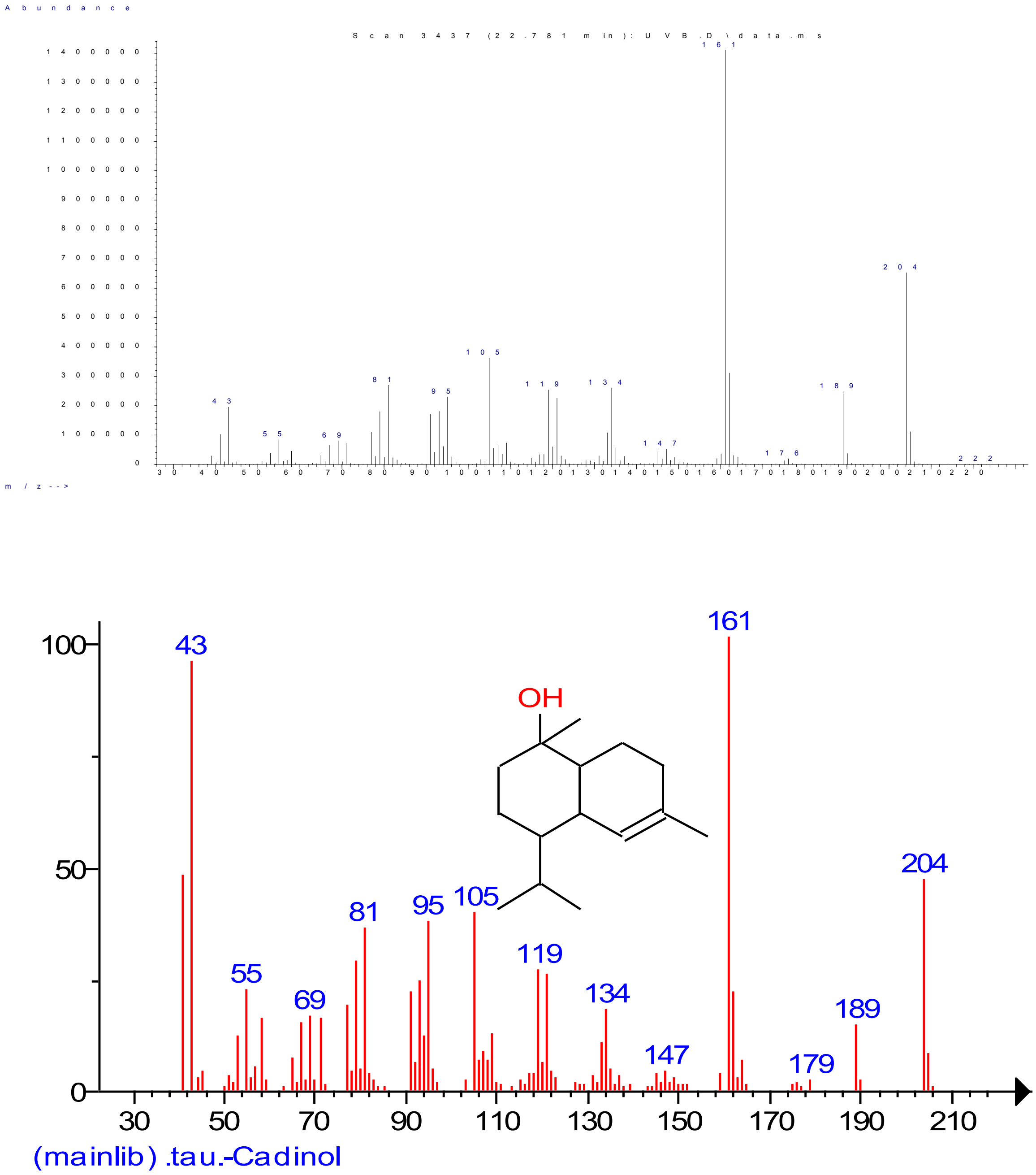

Figure 30: The experimental (up) and from the NIST database (down) MS spectra for α-cadinol identified from the GC-MS analysis of *O. basilicum* L. essential oil

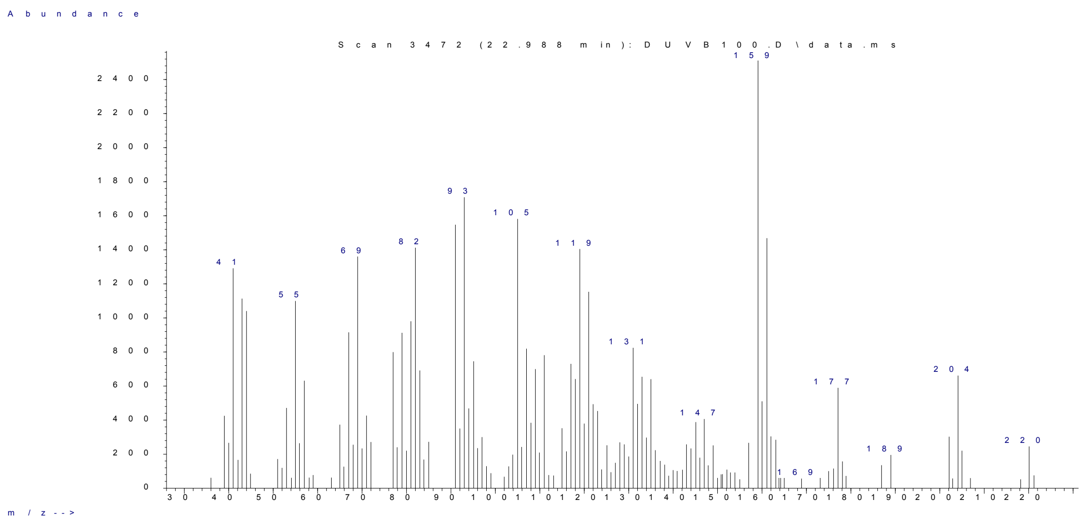

Figure 31: The experimental MS spectra for *Sesquiterpene oxide (isomer 1)* from the GC-MS analysis of *O. basilicum* L. essential oil

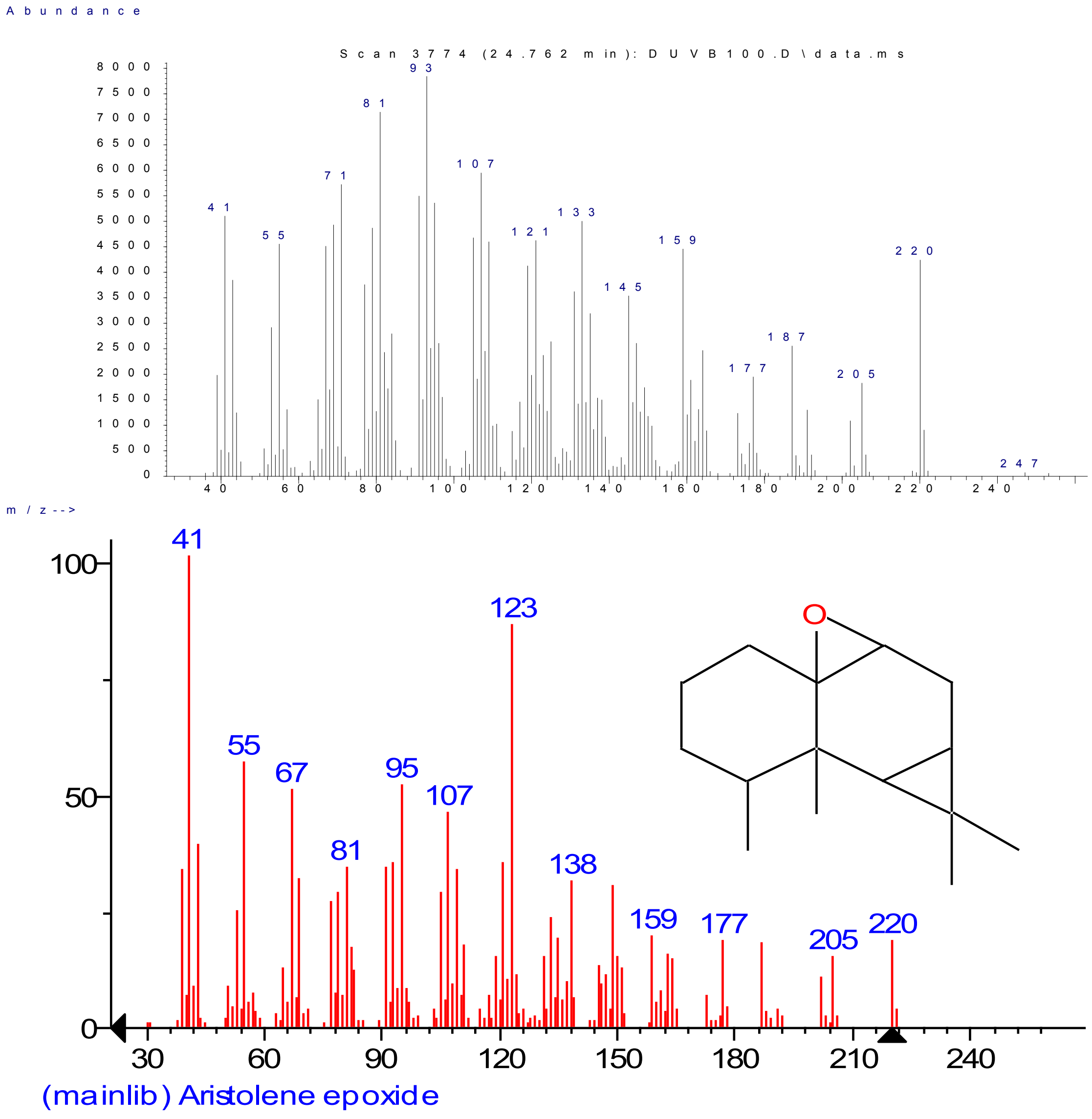

Figure 32: The experimental (up) and from the NIST database (down) MS spectra for aristolene epoxide identified from the GC-MS analysis of *O. basilicum* L. essential oil

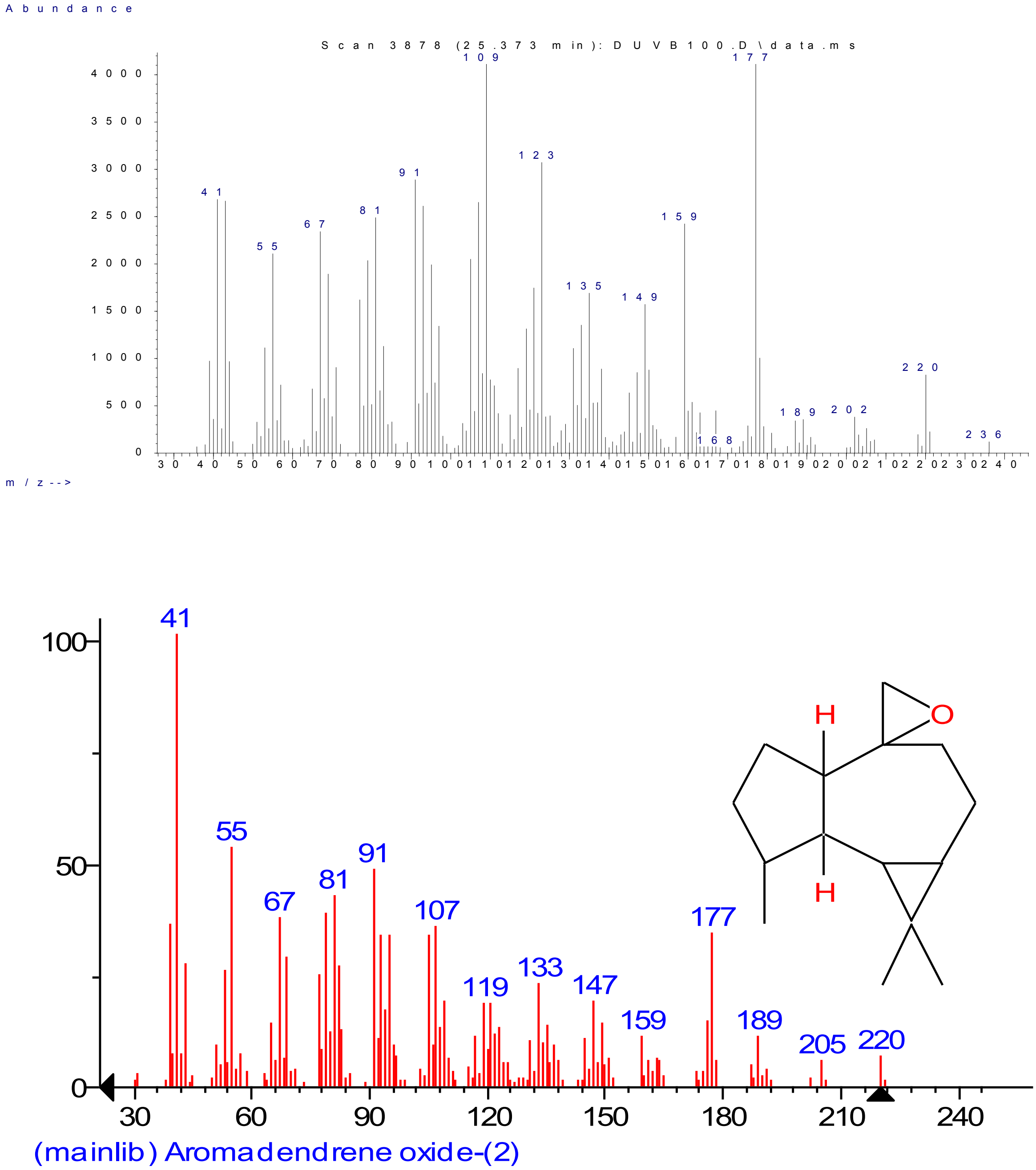

Figure 33: The experimental (up) and from the NIST database (down) MS spectra for aromadendrene oxide identified from the GC-MS analysis of *O. basilicum* L. essential oil
